# Communicating climate change and biodiversity loss with local populations: exploring communicative utopias in eight transdisciplinary case studies

**DOI:** 10.14324/111.444/ucloe.000064

**Published:** 2023-10-13

**Authors:** Dawud Ansari, Regine Schönenberg, Melissa Abud, Laura Becerra, Wassim Brahim, Javier Castiblanco, Anne Cristina de la Vega-Leinert, Nigel Dudley, Michael Dunlop, Carolina Figueroa, Oscar Guevara, Philipp Hauser, Hannes Hobbie, Mostafa A.R. Hossain, Jean Hugé, Luc Janssens de Bisthoven, Hilde Keunen, Claudia Munera-Roldan, Jan Petzold, Anne-Julie Rochette, Matthew Schmidt, Charlotte Schumann, Sayanti Sengupta, Susanne Stoll-Kleemann, Lorrae van Kerkhoff, Maarten P.M. Vanhove, Carina Wyborn

**Affiliations:** 1 Energy Access and Development Program (EADP), Wilmersdorfer Str. 122-123, 10627, Berlin, Germany; 2 German Institute for International and Security Affairs (SWP), Ludwigkirchpl. 3-4, 10719 Berlin, Germany; 3 German Institute for Economic Research (DIW Berlin), Mohrenstr. 58, 10117 Berlin, Germany; 4 Free University Berlin, Berlin, Germany; 5 WWF Colombia, Carrera 35 No. 4A-25 Cali, Colombia; 6 The Luc Hoffmann Institute, Rue Mauverney 28 1196 Gland, Switzerland; 7 Institute of Geography and Geology, University of Greifswald, Friedrich-Ludwig-Jahn-Str. 16, D-17489 Greifswald, Germany; 8 Equilibrium Research, 47 The Quays, Cumberland Road, Spike Island, Bristol, UK; 9 Commonwealth Scientific and Industrial Research Organisation, Building 101, Clunies Ross St, Black Mountain ACT 2601, Australia; 10 Technische Universität Dresden, Chair of Energy Economicy, Münchnerplatz 3, 01069 Dresden, Germany; 11 Department of Fish Biology and Genetics, Bangladesh Agricultural University, Mymensingh-2202, Bangladesh; 12 Open University of the Netherlands, Heerlen, Netherlands; 13 Biology Department, Vrije Universiteit Brussel, Brussels, Belgium; 14 Hasselt University, Centre for Environmental Sciences, Research Group Zoology: Biodiversity and Toxicology, Agoralaan gebouw D, 3590 Diepenbeek, Belgium; 15 CEBioS, ‘Capacities for Biodiversity and Sustainable Development’, Royal Belgian Institute of Natural Sciences, Operational Directorate Natural Environment, Rue Vautier 29, B-1000 Brussels, Belgium; 16 Fenner School of Environment and Society, Australian National University, Canberra, Australian Capital Territory 2601, Australia; 17 Department of Geography, Ludwig-Maximilians-Universität München, Luisenstr. 37, 80333 München, Germany; 18 Red Cross/Red Crescent Climate Centre, Anna van Saksenlaan 50, 2593 HT Den Haag, Netherlands

**Keywords:** transdisciplinary communication, climate change, biodiversity loss, knowledge co-production, postcolonial moments, local communities, local knowledge

## Abstract

Climate change and biodiversity loss trigger policies targeting and impacting local communities worldwide. However, research and policy implementation often fail to sufficiently consider community responses and to involve them. We present the results of a collective self-assessment exercise for eight case studies of communications with regard to climate change or biodiversity loss between project teams and local communities. We develop eight indicators of good stakeholder communication, reflecting the scope of Verran’s (2002) concept of postcolonial moments as a communicative utopia. We demonstrate that applying our indicators can enhance communication and enable community responses. However, we discover a divergence between timing, complexity and (introspective) effort. Three cases qualify for postcolonial moments, but scrutinising power relations and genuine knowledge co-production remain rare. While we verify the potency of various instruments for deconstructing science, their sophistication cannot substitute trust building and epistemic/transdisciplinary awareness. Lastly, we consider that reforming inadequate funding policies helps improving the work in and with local communities.

## Introduction

Climate change and biodiversity loss are concepts born and refined in global fora [1–4]. The respective discourses, which are dominated by concepts of the Global North [5,6], take place among scientists, politicians, civil servants and highly specialised segments of civil society.^1^ The concepts are based on the post-Enlightenment consensus that humans and nature follow different rationales [7,8]. Although the two concepts have different origins [9], both generate discourses seeking sustainability, trigger public policies and impact communities worldwide [10,11].

Anybody who has conducted transdisciplinary research or organised community-focused activities has probably noticed the stark asymmetries that occur when communicating topics related to climate change or biodiversity loss [12]. On the local level, such terms often encounter a lack of comprehension, as technoscientific representations of quantifiable causes and effects often remain alien to many local perspectives. Research on transdisciplinary science communication [13–15] demonstrates that we do not deal with a mere communicative gap but an entire cascade of tangible barriers in approximating ‘the local’ [8,16].

Nonetheless, thoughtful communication has a pivotal influence on successful research and joint project/policy implementation [17,18]. It is especially the creative co-production of knowledge that requires attention [19]. Ostrom [20] defines co-production as a process in which a common product is created through the contribution of actors from different origins. Accordingly, co-production can improve the effectiveness of research by linking it to community preferences and needs, which contributes to feasible solutions ([21], p. 251, 253), and co-production addresses the ‘relevance gap’ towards solving common problems [22]. Therefore, research instruments such as living-labs and citizen science, which test innovative sustainability approaches with relevant societal actors, have become more common [23–25].

Furthermore, policies often seek to include local actors through co-managing natural resources for conservation, mitigation and adaptation strategies [26,27]. Community-level responses are crucial for fighting global challenges [28]. Yet, neglecting to include communities during the various stages of a project creates a gulf that can hardly be bridged afterwards, ultimately eliciting failure to achieve the intended goals or even causing collateral damage [29]. For example, although the United Nations Conference on Environment and Development already acknowledged the value of indigenous and local knowledge for sustainable resource management back in 2002, bridging the communicative gap between different knowledge systems has not been adequately included in development or research programmes [30,31]. Local knowledge has large potential – for instance, species of agroforestry-systems can contribute to adaptation to droughts and, hence, to food security [32], and traditional knowledge about biodiversity indicators maps current developments accurately [33] – but it is mostly overlooked [28]. The success of climate and biodiversity goals depends on adequate communication and the agency attributed to local communities;^2^ there are still many gaps and barriers to address [34]. Notably, the land sharing/land sparing debate [35,36] sheds light on the role of underlying presumptions regarding global conservation policies.

In this article, we pick up these threads by examining how project teams actually communicate with local communities within the context of projects addressing climate change or biodiversity loss – and reflect on best practices and their own perception of diverging concepts.^3^ We showcase and analyse eight case studies that present such interactions during and after fieldwork in eight different countries (covering four continents). Each case study involves a specific set of approaches towards making global concepts accessible and connecting them to indigenous and local knowledge. We evaluate the case studies based on a set of eight indicators. They are derived from the critical literature on the communicative status quo [37,38] as well as Verran’s [39] communicative utopia of *postcolonial moments* projecting disruptions of epistemic power relations, which foster the co-existence and discursive construction of alternative knowledge systems. Thereby, postcolonial moments are also part of the endeavour to increase the agency of local actors. Hence, our indicators suggest where communicative processes should start connecting co-produced knowledge to sustainable transformation processes at the local level [40–43].

Method-wise, we draw learnings from an ex-post evaluation of the case studies based on our indicators. The case studies originate from our own fieldwork, which is why the approach amounts to a collective self-assessment and a peer-learning exercise. The narrative reflection of our own work, alongside the diversity of backgrounds and experiences among the authors, ensures a process that mimics an expert survey.

We aim for three contributions: first, we augment the academic discourse on communicating climate and biodiversity issues to the local sphere. Second, the article helps researchers and professionals in the field by providing communicative best practices and highlighting drawbacks to avoid. We wish to fundamentally challenge the idea of ‘science communication’ as it is currently being practised when communicating North–South. To this end, the study develops a model of the inner logic of progress towards postcolonial moments as well as tangible and straightforward insights on the benefit of various communicative elements; summing up the latter, we are projecting an eventual change in attitude. Third, we hope that the article stimulates a discussion among policymakers, project financers, and perhaps also among local communities on the role of, and requirements for good communication in the context of climate change and biodiversity loss projects. Our article supports, corroborates and deepens the call for research on community responses expressed by Washbourne et al. [28]. Accordingly, this article understands itself also as a call for a more profound preparatory training of Western(-ised) field researchers working in the Global South, who often assume that their concepts must be communicated and understood instead of scheduling enough time for comprehending and co-producing local knowledge and concepts.

The remainder of the article is structured as follows: the second section constructs a theoretical background for our work and presents our indicators of good stakeholder communication regarding climate change and biodiversity loss. Subsequently, the case study overview section presents and deconstructs the eight case studies according to various criteria, summarised in case study matrices (the Appendix contains detailed accounts of the case studies). The following section evaluates, analyses and discusses the case studies, based on the indicators defined in the second section. The section Stories of postcolonial moments portrays three examples of good communication to illustrate best practices. The Conclusion section sums up the article’s conclusions and offers policy recommendations.

## Theoretical background and indicator design

Climate and biodiversity are mostly approximated by technoscientific approaches such as computational models of geoscience, ecosystem analysis, the energy economy and any combinations thereof [59–63]; much of which is prominently covered in reports by the Intergovernmental Panel on Climate Change [64] and the Intergovernmental Science-Policy Platform on Biodiversity and Ecosystem Services [65].

These approaches allow for simplified shifts between global and local perspectives; however, reducing the discourse to models and numbers limits the factual scope of the analysis [66]. Quantifiable transformations that rely on de-contextualised approaches [67] suggest that analysis and solutions are objective; yet, such methods typically neglect social, political, cultural or local economic aspects [27,68,69]. Moreover, especially models that seek to approximate the regional level suffer from biases and insufficiencies in data and methods [59,70].

O’Lear [14] provides a critical perspective with a science and technology studies (STS)-oriented reflection of technoscientific ontologies of climate change. O’Lear finds that the dominant approaches, including the fixation on carbon indicators and their inherent cultural perception biases, obscure collateral damages on the local scale, ultimately causing the perpetuation of environmental injustice in the access to resources. O’Lear ([14], p. 2) links this phenomenon to Nixon’s [71] concept of ‘slow violence’: ‘Slow violence is not a movement, as are environmental justice and climate justice, but it is a concept that focuses attention on latent, gradual, and invisible negative externalities related to mis- or abuse of environmental resources and ecosystems.’

This aligns with a general marginalisation of local populations by implementing technoscientific environmental solutions without an integral drive towards mutual exchange and dialogue. For instance, state authorities can restrict access to natural resources in a protected area, a top-down action that threatens local communities’ ancestral livelihoods and their relation with land, or may criminalise local customs, products and economies [72–75]. Prominent examples are the effects of hydroelectric dams, mining or agro-industrial activities. Even if the impact of techno-centric top-down action is felt slowly, it is nonetheless violent; it is a gradual loss of agency and quality of life that may sometimes be unintentional yet could often have been prevented by appropriate transformation management. Hence, communication may also be the key to preventing slow violence from gradual change caused by secondary effects.^4^ Consequently, the epistemic, financial and political dominance of the protagonists leading the scientific and global policy process has resulted in predominantly technoscientific approaches and solutions that often fail to consider the abundant sociological and anthropological research covering the same domains [76,77]. Such bias is deeply rooted in the history of knowledge production, and scholars rarely explore ‘the ways in which science can be conceived as being composed of “travelling narratives”’ ([78], p. 273). Hence, a critical reflection on the origins of scientific presumptions is necessary. Answering James Clifford’s [79] question, ‘How do theories travel among the unequal spaces of postcolonial confusion and contestation?’: between social media and interdisciplinarity, attention should be paid to circulating narratives transporting fragmented rights and wrongs.

Accordingly, changing the perspective towards a deeper understanding of the perpetuation of unsustainable lifestyles and its overcoming may be crucial, such as proposed by Hulme ([80], p. 335): ‘The challenge of responding to climate change is to turn our gaze away from making firmer, newer, or more integrated scientific knowledge and instead to ask why enacting directed change is so hard to accomplish. It is less about asserting firmer facts about the world or constructing less uncertain projections of the future. Rather, it is more about cultivating appropriate public spheres of contestation and deliberation about multiple and diverging worldviews, beliefs, and value systems.’ Hulme emphasises the limited powers of human agency due to the complexity and uncertainties prevalent in climatic systems. According to him, the fusion of method-based scientific and holistic local knowledge – something amounting to a knowledge–perception–narrative nexus – might close knowledge gaps despite different worldviews. Is it probably more than a communicative gap, due to ‘the problem that the difficult normative dimensions of the relationship between knowledge, values, and action have not been sufficiently attended to’ ([80], p. 334). This is precisely the path on which we would like to follow up.

The literature covers different examples of bridging communication gaps between diverse knowledge systems and perspectives, such as Mar Delgado-Serrano et al. [81] for Latin America and Hill et al. [82] for Australia. However, Verran’s [39] work on postcolonial moments may be the most powerful description of the necessary paradigm shift. In the context of an encounter between Western scientists and Aboriginal landowners for a workshop on fire regimes, in which local knowledge was met with incomprehension and ignorance, Verran highlights the importance of being aware of the various biases towards local knowledge. She ([39]: p. 730) describes *postcolonial*^5^ *moments* as disruptions to ‘power relations characteristic of colonising’, involving ‘both, making separations, and connecting by identifying sameness’; this ‘sameness’ ‘is not a dominating universalising’, but it ‘enables difference to be collectively enacted’. Postcolonial moments happen when competing knowledge systems clarify similarities or disagreements in new ways without alienating each other, fostering mutual understanding and interest in a discursive construction of each other’s world. This process requires allowing enough time for reciprocal approximation and dialogue towards postcolonial moments of understanding (cf. [85] on ecological reflexivity as a way to reframe sustainability in the context of maladaptive modern institutions).

Why do we consider such postcolonial moments desirable, and what can be gained from them? Assuming that creating an effective communicative level between different knowledge systems is an extraordinary challenge, it is difficult to find reference points for a genuinely non-hierarchical exchange. The concept of postcolonial moments offers identification with a common goal based on the generalisation of comparable practices to achieve this goal. The remaining tension in the construction of sameness can be bridged by the storytelling of practical examples that would fit generalisations, supported by mutual respect for differences. This is where we locate the possibility of theorising jointly, pointing out differences and naming similarities. While academics working in the Global South often find themselves in the camp of colonial traditions, the pursuit of postcolonial moments offers the chance to break traditional power relations and reallocate agency. The latter increases the options for co-production by respecting differences and acknowledging the common colonial past. In the words of Verran ([39], p. 757), postcolonial moments offer ‘a starting point for non-hierarchical knowledge exchange between different knowledge systems’. In this sense, the concept connects to creative co-production [20], which has been operationalised by Durose et al. [22] towards closing the ‘relevance gap’.

Constructing a discursive space for such exchange on equal terms requires reflecting on power relations, time and space for communication [86,87].

Therefore, and building on the theoretical framework established above, we define a set of indicators of good stakeholder communication regarding climate change and biodiversity loss (see Table 1). These criteria reflect the settings of a *good* communicative process as suggested by the interdisciplinary literature covering the co-production of knowledge. (1) Implies the (sufficient) allocation of time and human resources to the communicative process [13,86]; (2) calls for the reflection of space permeated by power relations in which knowledge production takes place [86,87]; and (3) refers to the unequal access to (natural) resources by the different actors involved [88,89]. (4) Calls for deconstructing technoscientific concepts and recontextualising problems and solutions connected to ‘the local’ [14,16]. The de-hierarchisation of communication (5) requires sensitivity from the involved parties as well as a clear and respectful inner attitude [14], which can also be fostered by the inclusion of local narratives (6) [81]. This may lead to an appreciation of diverging world views, beliefs and value systems (7) as well as decentring knowledge and value systems (8) [39]. These eight elements are sometimes partially realised, adding to gradually better communication, possibly allowing for a postcolonial moment.

**Table 1.** Indicators of good communication

#	Indicator	
1	An acknowledgement of the role of communication and the resources it requires	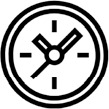
2	An analysis of the local and intra-project power relations	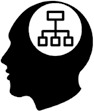
3	A reflection on environmental injustice	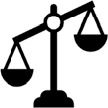
4	A deconstruction of technoscientific concepts	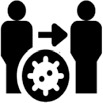
5	A de-hierarchisation of communication	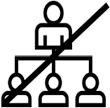
6	An inclusion of local narratives	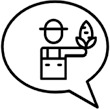
7	An appreciation of diverging worldviews, beliefs and value systems	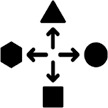
8	A decentring of knowledge and value systems	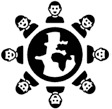

## Case study overview

This section introduces our eight case studies (see Table 2).^6^ They originate from eight different countries in four different regions: Brazil and Colombia in South America, India and Bangladesh in South Asia, Tanzania and Egypt in Africa and Germany and the British Isles of Scilly in Europe, each one covering a distinct communicative process. The Appendix provides detailed narrative accounts of each case study, whereas Table 2 summarises the case studies, Table 3 provides an overview of the communication in each case study, and Table 4 contains central successes, drawbacks, learnings and surprises in the case studies.

**Table 2.** Case study overview

Title	Communicating climate change: what’s the forest worth?	Co-producing and co-learning climate adaptation strategies in biodiversity conservation: lessons from Colombian protected areas	Communicating climate change in the Indian Sundarbans	Communicating grassroot stakeholders: climate change and biodiversity crisis in coastal Bangladesh	The Aswan DESIRE Workshop on socio-economic impacts of RES in MENA countries	Ecosystem Services as a rallying concept in multi-stakeholder workshops on biodiversity management and conservation	Dissidence and sabotage to redress scientific bias in communicating desirable coastal land management futures	Fieldwork experiences from climate change adaptation research on the Isles of Scilly
Region	South America		South Asia		Africa		Europe	
Location	Amazon rainforest, Brazil	Various protected areas in Colombia	Mousuni Island, India	Shyamnagar Upazila, Bangladesh	Aswan, Egypt	Lake Manyara Basin, Tanzania	Baltic Sea, Germany	Isles of Scilly, United Kingdom
Context *(What is the larger context of the case study?)*	Interdisciplinary research project on climate change and land management. The activity aimed at assessing carbon stocks, analysing knowledge production, and providing indigenous people with data for REDD+ projects	Interdisciplinary research project on how to strengthen protected-area managers’ capacities to anticipate and respond to climate change and to rethink conservation and management strategies for climate adaptation	Research project on the effects of water-related hazards on the vulnerability of islanders to climatic events. Analysis of the adequacy of institutional support for locals who lost faith in gov. support and engaged in maladaptation practices	Research project on trends in aquatic ecosystems of the coast of Bangladesh. Investigation of community perceptions on changes in the ecosystem, biodiversity and their impacts	Capacity-building project for higher education institutions in teaching students and young professionals in the MENA region on evaluating the socio-economic impacts of renewable energy/energy efficiency	Multi-disciplinary research initiative-project to support the development of a decision-support system for integrated water management and for assessing priority ES	Interdisciplinary research project on climate change and coastal land management. Key topic: evaluation of coastal protection scenarios based on managed realignment compared to conventional hard defence	Research project to analyse the role of social capital and community resilience in the context of climate change adaptation
Duration of the case study event (without interviews)	4 weeks field trip and 1 day presentation	40 days with various workshops 28 individual interview sessions	6 days with workshops 120 individual interview sessions	60 workshops of 3 hours each	1-day workshop	6 days, split into two workshops	1-day World Café and 21 expert interviews	35 interview sessions, split over 9 weeks 1-day workshop
Focus area	Mitigation (REDD+)	Adaptation and conservation (various)	Adaptation (extreme weather)	Adaption (responses to biodiversity loss)	Climate change mitigation (renewable energy)	Adaptation (ecosystems)	Mitigation (CO_2_ storage) and adaption (sea level rise, extreme weather)	Adaptation (sea level rise)

**Table 3.** Communication within the case studies

Location	Amazon rainforest (Brazil)	Colombia	Mousuni Island, (India)	Shyamnagar Upazila (Bangladesh)	Aswan (Egypt)	Lake Manyara Basin (Tanzania)	Baltic Sea (Germany)	Isles of Scilly (United Kingdom)
Duration (incl. preparation)	6 months	36 months	6 months	12 months	6 months	12 months	18 months	18 months
Context and intention *(To which communic. does the case study refer?)*	Field trip with community participation and a presentation for indigenous leaders Intention: knowledge extraction and, later, dissemination	Multi-stage, dialogue-based activity series with stakeholders Intention: dissemination, transitioning to co-production of knowledge	Multi-stage primary survey with focus-group discussion, interviews, and workshops with different stakeholders Intention: extraction transitioning to co-production of knowledge	Household surveys and focus-group discussions Intention: extraction of knowledge	Local stakeholder workshop with talks and discussions for dissemination and identification of deficits Intention: dissemination of knowledge	Two multi-stakeholder participatory workshops, surveys, and field visits Intention: dissemination, transitioning to co-production of knowledge	Multi-step process to assess pre-formulated scenarios with semi-structured interviews and World Café Intention: Co-production and evaluation of scenarios; Dissemination of knowledge	Multi-stage fieldwork with quantitative surveys, semi-structured interviews, and participant observation Intention: extraction of knowledge
Recipients	Indigenous leaders; indigenous youth during the field trip	Primarily protected-area managers. In some stages, local NGOs and communities	Local communities and NGOs, government officials at the village and district level	Community members, directly and indirectly, dependent on aquatic systems	Local leaders, civil society representatives, journalists, business owners	Local authorities, NGOs, pastoralists, smallholder farmers	Experts (for interviews and World Café) and interested public World Café and focus groups)	The local population, local authorities, NGOs, landholders, experts, and media,
Instruments	Presentation of results with carbon deconstructed to ‘energy’ and REDD+ mechanisms as a contract Visualisations with cartoons and comparisons to everyday life experiences of local indigenous leaders Common field trip with daily discussions	Interlinked five-stage participatory dialogue with varying degrees of stakeholder involvement Sequential workshops with different stakeholders In-depth interviews Visualisation of participant responses with diagrams and cartoons	Trust building Awareness raising (documentaries, videos, pamphlets in the local language) Participatory rural appraisal techniques to represent local resources Interactive construction of historical timelines Discourse and narrative visualisation Questionnaires and Participant observation	On-site literature survey of local concerns (Key-informant) Interviews Narration-based deconstruction of biodiversity in interactive sessions Focus group discussions Questionnaires design of a tailor-made questionnaire Ranking of aquatic resources	Lecture-style talks and presentations Brief discussions Feedback survey asking for the participants’ opinion	Facilitated brainstorming in group discussions, drawing from experiences Collective stakeholder analysis (interest–influence matrix) Problem/solution trees Drawing of community-specific maps Collective field visits Videos of testimonies about workshops	Expert interviews formed the basis of one ‘stakeholder-based scenario’ World Café participants were asked to comment, reject or approve the scenarios Scenarios visualised possible coastal evolutions in different time steps Evaluation of scenarios’ rationale	Fieldwork spread over different seasons Early media announcements (local radio, websites) Interviewees could decide on the ‘terms’ of the interview Public discussion of research results Participant observation Climate change deconstructed to hazards and impacts

**Table 4.** Learnings and drawbacks in the case studies

Location	Amazon rainforest (Brazil)	Colombia	Mousuni Island, (India)	Shyamnagar Upazila (Bangladesh)	Aswan (Egypt)	Lake Manyara Basin (Tanzania)	Baltic Sea (Germany)	Isles of Scilly (United Kingdom)
Ex-ante challenges *(Which initial challenges did the communication face?)*	The communication concept was not aiming at mutual exchange but at unilateral communication of scientific facts	‘Accommodating ecological change’ conflicts with present rules to maintain ecological attributes Climate change is regarded solely as an exogenous, technoscientific problem, separated from governance/decision-making	Limited awareness of the (potential) connection between mangrove depletion and deforestation in general to the increasing intensity of extreme climate events Lack of political appetite and capacity among government authorities to engage in conversations about climate issues	Stakeholders used to top-down approaches by project managers and governmental representatives	Limited communication between the European team and local organisers Limited interdisc. understanding of participants Participants are unfamiliar with participatory formats	Implementation of the ‘evidence-informed’ approach tedious and complicated Indicator-based communication and ES often too complex for communication Audience varies unpredictably between workshops	‘Managed retreat’ often provokes resistance Co-Design is difficult to integrate in natural-sciences-dominated projects Stakeholder preferences are difficult to include in quant. modelling	Scepticism towards UK-based ‘experts’ Heterogeneity of stakeholder perspectives and preferences Varying population and weather patterns between different seasons
Special achievements *(What worked out especially well?)*	Joint data generation allowed for insights into the ‘making of’ science Novel data obtained that would not have been available without this collaboration	Construction of a common ‘native’ narrative Past experiences and reflecting on uncertainty and ecological transformation helped reframing assumptions and move from reactive management to anticipation	Interactive construction of timelines and visualisations helped to tap and access local knowledge and establish a common ground on challenges and need for biodiversity preservation Established an initial understanding of the inter-dependency between maladaptation practices and climate vulnerability	Using local facilitators talking local dialect referring to a locally found habitat; instead of ‘biodiversity’ use of concrete examples of aquatic fauna	Large number of attendees Project coverage online and in newsletters 70% ‘very good’ or ‘good’ feedback responses	Comparative analysis of literature and stakeholder perceptions worked out Locally respected facilitators in own language helped gaining trust and access Community mapping was the most attractive tool in terms of ownership and participation	Using the concept of ‘land management’ helped to move the focus away from coastal defence and enabled debate on alternatives	Successful deconstruction of climate change due to local narratives (sea level rise/storms) Including a variety of stakeholders across seasons reduced biases Transparent approach increased trust
Drawbacks and difficulties *(Which problems persisted?)*	A technoscientific representation of climate change as a ‘problem to be measured’ prevailed among the scientists and obstructed an exchange on equal terms	A natural disaster forced the organisers to cut two of the planned four workshops	Links between global phenomenon and climatic events; between decreasing biodiversity and increasing vulnerability on the islands were not entirely established within the limited time frame	Communic. of biodiversity concept was only partially successful Local units were largely unknown The multitude of local names for single species led to confusion	No translation available for European researchers Diverging objectives of organisers (dissemination vs. participation) Monopolised discussions by speakers and elites Participants refused an interdisiplinary discourse	SES were too complex for time frame and target audience Participants expected ‘quick solutions’. Economic valuation of ES could not be realised Struggle for resources amongst participants	Tight control of the participatory process led to unplanned bottom-up responses, where some participants rejected the steered process to question the scenarios and their underlying assumptions and reclaim control of the evaluation process	High inter-seasonal variability of locals (e.g., second-home owners and busy tourism-sector affiliates are only available in summers) made it difficult to capture ‘all’ voices
Surprises *(Which unexpected developments or insights resulted?)*	Local leaders were more interested in methods (e.g., how to determine the price of emissions to be certified) than policies	The communication was first hierarchical, despite extensive consultation during development and a sincere commitment to co-production	High willingness of the inhabitants to take part in participatory discussions and finding solutions together to increase resilience to future climate events	Expectations of concrete help from the research project regarding biodiversity loss Each species had two to four local names	A higher share of female participants than expected Some participants engaged to create business networks with European project partners	Pastoralists acknowledge differentials in grass quality but avoid discussing overgrazing Pastoralists seemed rather unconcerned about the drying of the (saline) lake	Protesting participants created their own dynamics by reshuffling the rules of evaluation and by constructing a scenario that fitted their preferences	High awareness of the islands’ historical sea-level changes Despite the scepticism towards UK-based ‘experts’, the (German) researcher was welcomed by the stakeholders
Main learnings *(What can we learn with regards to the communic process?)*	Obstacles from persistent diverging interests of researchers and stakeholders Co-design of topics is key to successful transdisciplinary research No ‘objective’ way to discuss climate change Climate change images are still not disentangled from colonial settings and socioeconomic imbalances	Local knowledge on adaptation can be as important as science for informing decisions Climate adaptation connects to various values and chances Communic. should highlight co-benefits and immediate management opportunities rather than potential future approaches	Local knowledge needs to be systematised and included in policy discourses Potential points of Conflict and awareness of the local dynamics are important for researchers/external agents Regular communic. on global climatic events is necessary to take local communities onboard for adaptation	Assessing the local knowledge-base and using local languages is necessary to work with the community on these challenges	Necessity to harmonise organisers’ objective Take measures to enforce active participation of all attendees Include stakeholders of different academic backgrounds	Impact limited to local awareness-raising Mixed methods, tangible and rallying concepts as well as examples from everyday life are useful Small groups better than plenary to overcome social control and hierarchies Respected locals and civil-society intermediaries crucial for process and legitimacy	Perception, preference and rationalisation gaps between science, policy and local population Co-production requires balancing participation and control Scientists need to be persuaded to use co-design Funding agencies need to give more flexibility to use exploratory co-production	Local experiences and awareness are necessary to allow deconstructing concepts Biases can be reduced by including non-dominant stakeholders and extending the time frame over different seasons Transparency is crucial for gaining trust and participation

Each case study is an ex-post empirical observation of a communicative process with a local community or local experts. All dialogues happened within research frameworks or, in one case, capacity-building projects that did not explicitly investigate communicative processes. Instead, the researchers developed their communication strategies solely to fulfil their projects’ objectives without explicitly considering the topics addressed by this study. Therefore, the variety of contexts and communication instruments provides a valid basis for analysing the determinants of successful communication and extracting conclusions and recommendations that may be extrapolated.

In the Brazilian Amazon, deep carbon measurements in the context of indigenous REDD projects (Reducing emissions from deforestation and forest degradation in developing countries) led to the case study ‘Communicating climate change: what’s the forest worth?’. In Colombia, extensive fieldwork on the management of protected areas provided the basis for the case study ‘Co-producing and co-learning climate adaptation strategies in biodiversity conservation: lessons from Colombian protected areas’. The case study ‘Communicating climate change in the Indian Sundarbans’ originates from a remote area of northeast India, where climate change was known as a term though not as a concept. Similarly, the case study ‘Communicating grassroot stakeholders: climate change and biodiversity crisis in coastal Bangladesh’ reports from the experience of investigating the consequences of aquatic biodiversity loss in Bangladesh. From Tanzania, the case study ‘Ecosystem services as a rallying concept in multi-stakeholder workshops on biodiversity management and conservation’ covers the usage of an innovative toolbox for stakeholder communication. Moving indoors, we also have two case study examples of more conventional communications: the case study ‘The Aswan DESIRE Workshop on socio-economic impacts of RES in MENA countries’ from Egypt and the case study from the German Baltic Sea coast, ‘Dissidence and sabotage to redress scientific bias in communicating desirable coastal land management futures’. Our last case study, ‘Fieldwork experiences from climate change adaptation research on the Isles of Scilly’, covers the experience of extensive fieldwork on a British archipelago on local climate change impacts.

## Analysis and discussion

Here we discuss the case studies. We start by assessing the case studies based on the eight indicators defined in theoretical background and indicator design section. Subsequently, we discuss to which extent the indicators have proven valid measures of communicative achievements. We then move forward to identifying best practices and their determinants among the case studies.

### Assessing the case studies

We start with an individual assessment of each case study. Table 5 summarises our results: based on the case studies’ approaches to communication and their respective fulfilment of our indicators (cf. Tables 3 and 4), we mark whether the indicators (cf. Table 1) were not (sufficiently) fulfilled, partially fulfilled or were strongly fulfilled. We evaluated an indicator as ‘not (sufficiently) fulfilled’ if the case study description does not cover efforts towards fulfilling the respective indicator, ‘partially fulfilled’ if the case study exhibits some attempts at fulfilling the indicator although with limited effort or success, and ‘strong fulfilment’ if the case study showcases major efforts and success towards the respective indicator.

**Table 5.** Indicators of good communication in the different case studies

Case study indicator	Amazon Rainforest (Brazil)	Colombia	Mousuni Island (India)	Shyamnagar Upazila (Bangladesh)	Aswan (Egypt)	Lake Manyara Basin (Tanzania)	Baltic Sea (Germany)	Isles of Scily (United Kingdom)	
1. Acknowledgment of necessary resources								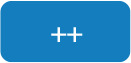	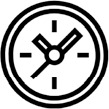
2. Analysis of power relations									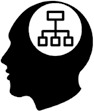
3. Reflection of environmental injustice									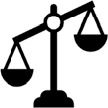
4. Deconstruction of technoscientific concepts	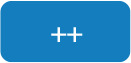	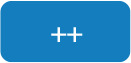							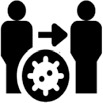
5. De-hierarchisation of the communication				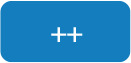				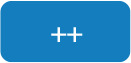	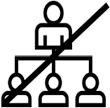
6. Inclusion of local narratives				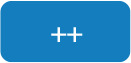				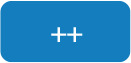	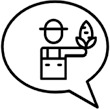
7. Appreciation of divergence				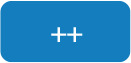				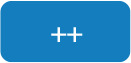	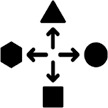
8. Decentring knowledge and values									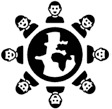
*Postcolonial moment conceivable*									

Note: an empty cell (red) marks no significant fulfilment, ‘+’ marks fulfilment (light blue), ‘++’ marks strong fulfilment of the indicator (dark blue).

In the case study from the Amazon rainforest (Brazil), researchers communicated with indigenous people to gather research data on deep carbon and, in a second step, to provide the communities with the respective data for REDD+ negotiations. The second goal was formulated after a sound reflection of power asymmetries and environmental (in-)justice in compensation schemes. However, the research project neither foresaw knowledge co-production nor local knowledge-transfer towards the researchers. On the contrary, the communication was limited to a unilateral presentation of scientific facts by deconstructing carbon towards energy. As the community perceived the communication as a mere top-down event, indigenous leaders remained indifferent to the research results, despite their explosive political nature. Instead, they showed interest only in practical matters such as carbon pricing.^7^ During a joint field trip with indigenous youth, it became clear that the technoscientific conceptualisation of climate change (i.e., *something to be measured*) prevented a more profound knowledge exchange.

In the Colombian case study, most indicators of good communication were eventually fulfilled. Extensive consultation during the project and translations of the relevant material to the local language contributed to the communications’ de-hierarchisation, which was also apparent during the workshops. The project set out to deconstruct the technoscientific framing of climate adaptation and biodiversity conservation by creating engagement between belief and knowledge systems, analysing the institutional factors shaping decision-making, eliciting stakeholders’^8^ past experiences with change. They included local narratives to work with ‘future proofing’, drawing from shared ideas about the benefits for protected areas and built a baseline of climate-change-related knowledge. The researchers have shown a deep appreciation of the local by mentioning that ‘local knowledge on adaptation can be as important as science for informing decisions’. The team has proven diligence by adjusting the resources allocated for each workshop individually and timing, location and context.

Regarding the case study from the Indian Sundarbans, researchers aimed to study the vulnerability of local communities to climate-related hazards. The scientists claimed to transparently communicate this goal and the purely scientific nature of the project. The technoscientific approach was deconstructed by visualising the relationship between the destruction of the mangroves and extreme weather events and personalising the impact on local communities, especially women, over time. A joint resource-mapping achieved trust-building and the inclusion of local narratives. It was followed by the joint construction of a historical timeline, which demonstrated extreme weather events and subsequent mangrove depletion over time. An appreciation of divergence is evident from the learnings: the researchers concluded that local knowledge should be better assessed and included in climate adaptation plans and that scientists should research local communities’ socio-economic and cultural characteristics beforehand. The study also found that maladaptation practices resulted from information asymmetry and a lack of agency and alternatives. However, the researchers did not anticipate the resources necessary for sharing information on how global climate change and biodiversity loss exacerbate the frequency of extreme events on these islands. While the researchers reacted with successful improvisations, they could not entirely deconstruct technoscientific concepts.

The Bangladesh case study covers a long-term investigation of community perceptions on changes to biodiversity, productivity, livelihood and adaptation responses. The scientists were aware that stakeholders are accustomed to a top-down approach, which is why they invested time and instruments in the de-hierarchisation of communication and the deconstruction of the technoscientific concepts. This was reflected in the intuitive nature of the questions, which covered personal experiences that exemplified the impacts of climate change and biodiversity loss with changes in livelihood and their suspected reasons. At the beginning of each dialogue activity, the team would initiate interactive storytelling using local dialects and examples from the surrounding ecosystems. They aimed to include local narratives to encourage broad participation while further de-hierarchising the discussion and allowing the participants to create their own biodiversity narratives through their own stories and scenarios. The scientists emphasised a substantial communicative gap between scientific understanding and common ‘problems’, which could only be bridged by a clear understanding of the local perspectives. This case study fulfils all indicators striving towards a postcolonial moment.

The Egypt case study depicts a conventional communication, where project results were disseminated in a top-down style. Thus, the communication was overly hierarchical and did not break through the firm social hierarchies among the attendees. The researchers have actually assessed the local and intra-project power relations very well; however, the considerations did not affect the workshop planning. This resonates well with the non-acknowledgement of other requirements, such as interpreters. Technoscientific approaches were not deconstructed or connected to local narratives apart from employability and the local economy. More advanced stages of communication – such as a decentring of systems – were not pursued. However, it is noteworthy that these shortcomings occurred primarily because of differences between the European team and the local academics, who organised the event mostly by themselves. Hence, the pivotal communication to assess might not be the one taking place during the workshop but the one related to the organisation process. However, the final audience had a positive impression of the workshop and was satisfied with the results. Thus, there may be significant untapped potential in the community for further communication efforts.

The case study from the Lake Manyara Basin (Tanzania) shows a highly sophisticated approach towards the co-production of a decision-support system. The researchers used a multitude of communication techniques to capture and include local views, supported by simultaneous language interpretation. Also, using a co-produced stakeholder analysis, the researchers aimed to assess and include local power relations. They were open to learning from the local population, and their evidence-based approach aimed at integrating mainstream perspectives and local knowledge into one structure. However, despite their multitude and sophistication, the deconstruction of technoscientific knowledge was only partially successful: the target audience did not fully comprehend the (North and South) researchers’ presentations and group exercises on social-ecological systems (SES) (notably, the valuation and flows of ecosystem services [ES]). The local community’s tendency to expect ‘quick solutions’ from the researchers indicates that the implication of local scientists and colleagues from elsewhere in the Global South may not suffice to de-hierarchise the communication and lead to a postcolonial spirit.

The Baltic Sea case study (Germany) illustrates a traditional approach to stakeholder engagement in science-dominated projects. The project team engaged experts and the local community in a strongly steered communication about science-driven scenarios on coastal land management. To satisfy the somewhat contradictory expectations by the funding agency (a strong emphasis on specific modelling approaches while also demanding participatory settings), scientists originally planned to control the agenda, the proposals to be considered by stakeholders and the evaluation methods rather than to yield power to the involved stakeholders, engage in true co-design and create a balance between both sides. Although the project invited different voices in different participation formats and included visualisation instruments, stakeholders could not shape the project. The discussion remained a hierarchical scientist-to-expert and local population approach. During a session of interactive group discussions, a group of stakeholders in strong disagreement with the scenarios presented rejected the top-down rules of evaluation to achieve their own goals and bring their preferences to the fore. This spontaneous bottom-up response contributed to a delayed appreciation of divergent views, fed internal critiques of the conventional distribution of power within the communication, and the project team’s deconstruction of the technoscientific language. However, this could not fundamentally alter the project’s predetermined conditions and power structures.

In the Isles of Scilly case study (United Kingdom), interviews about climate change adaptation were conducted individually. They included non-dominant voices, and interviewees could decide on the terms of the interview. Thus, the communication could be de-hierarchised, and a multitude of local narratives – also marginal ones – were emphasised. These efforts also showcase the non-prescriptive role the researcher takes; they learn from the participants in their chosen settings, thus appreciating their perspective and system. Also, through extensive trust-building, the researcher presents themself as a mediator of diverging perspectives and values. Climate change was deconstructed to hazards and impacts, although the islanders’ widespread awareness of climate-change issues might have pre-empted this effort. Notably, the case study was spread over multiple seasons, significantly contributing to trust-building and, hence, the communication’s success.

### Discussing the role of the indicators

**Figure 1:**
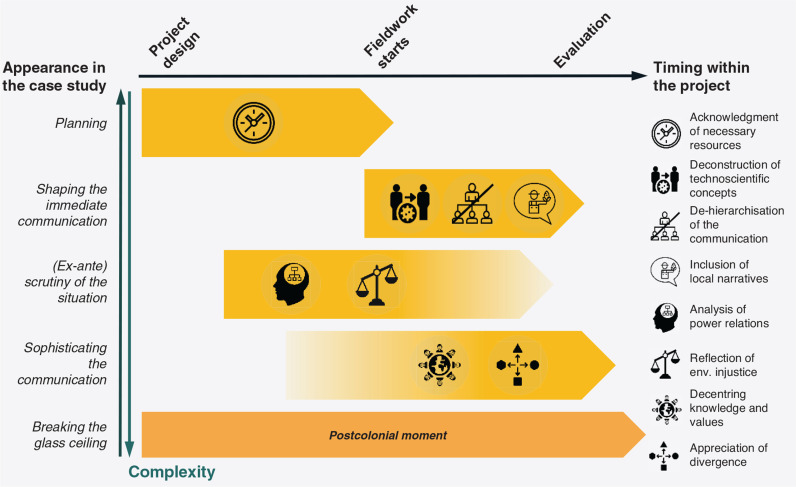
Illustration of the proposed structure of indicators and their timing within the project.

The indicators relate to different phases of the project process (see Fig. 1). An acknowledgement of necessary resources is required *before the project starts* (i.e., when designing the project). Analysing power relations and reflecting on environmental justice relate to the underlying theoretical framework and require interdisciplinarity; these aspects are relevant when exploring the region/community *before the actual fieldwork starts*. Having some idea about these concepts is a precondition for the de-hierarchisation of communication, which – alongside a deconstruction of technoscientific approaches and the inclusion of local narratives – occurs *during communication*. An appreciation of divergence and the decentring of systems arise from the participants’ mindset *during the knowledge exchange* and *during the evaluation* of results.

In our case studies, a comprehensive reflection of frame-conditions (power and justice) or a successful de-hierarchisation occur less frequently than the inclusion of local narratives or a deconstruction of the respective technoscientific approaches. In other words, the ‘on-the-spot’ shaping of the immediate communication seems more widespread than ex-ante scrutiny of the situation. Consistent with the structure postulated in the previous paragraph, the indicators for the further sophistication of the communication to happen during and after the knowledge exchange (i.e., the decentring of belief and knowledge systems and the appreciation of divergence) appear even less frequent; we see them mostly in case studies that already fulfil the other indicators.

Thus, we anticipate an idiosyncratic structure of advancing communication towards postcolonial moments; the structure’s order adheres to the social and introspective effort required to fulfil the indicator instead of its actual timing (Fig. 1). It disembogues into a general divergence of timing, logic and complexity. Considering the resource requirements (e.g., time, human resources, inviting stakeholders) is both the earliest and most obvious action. When approaching fieldwork, shaping the immediate communication^9^ is an easily recognisable need for achieving project results. Scrutinising situations and circumstances must (primarily) happen beforehand, but they require more active efforts by researchers and practitioners and a mature perception of the communicative process. Further sophistication, however, requires more than careful planning at every stage – it demands an inner, personal effort driving the project: powerful project professionals and academics need to lay down their guard and their widespread beliefs of hegemony concerning scientific knowledge as the panacea or sole possible framing of reality to start learning from and with local communities.

Furthermore, only a few case studies made efforts to include a reflection on social-environmental injustice explicitly. This observation is not necessarily at odds with our suggested framework, but it may lead to a caveat. It is conceivable that analysis of the power relations and environmental injustice are substitutes rather than complements. To move the communication forward, it is not essential to scrutinise all aspects if the communication has risen to a level where the participants feel confident enough to voice their concerns about secondary effects and slow violence. On the contrary, the lack of reflexivity towards environmental injustice in our case studies confirms that even projects with sophisticated communications tend to focus on interpersonal relations while neglecting overarching mechanisms within the human–nature interaction, which are increasingly shaped by criteria of capitalist exploitation [89–91].

### Identifying and discussing determinants

This subsection reflects on the insights acquired hitherto and discusses selected elements that enable successful communication. While the previous subsection focused on a more abstract, conceptual level, this part covers a more tangible approach towards assessing the case studies. It relies on the various details indicated in the project matrices (see Tables 3 and 4) in addition to the assessment made in Table 5. For many, sophisticated techniques (including visualisations) that break down technoscientific concepts may be the most intuitive approach towards designing ‘proper’ communication with local communities. Indeed, all (but one) of our case studies rely on such methods, ranging from problem-solution trees to drawing imagery to conducting interviews. While the case studies suggest that respective methods are necessary to enable a common understanding, their comparison showcases that they are neither sufficient nor can they take a ‘one size fits all’ form. The Bangladesh case study, which fulfils most indicators, contains only a single oral approach to deconstruction and abstains from any more sophisticated elements (such as visualisations). In contrast, the example from the Baltic Sea shows that visualisation alone do not guarantee successful communication, especially if their underlying normative premises are not openly discussed and negotiated with participants.

The case study from Tanzania deserves special notice in this regard. Among all case studies, it uses the most sophisticated toolbox of instruments during communication. However, they were only partially successful in deconstructing science, as some topics remained opaque to the audience. Furthermore, the community expected ‘quick solutions’ from the project team. The latter hints at the approach’s shortcoming in de-hierarchising communication and transforming it into a genuine, decentring process of exchanging knowledge and beliefs between both sides. Instead, and although half of the scientists were from the Global South, the local community continued to perceive a top-down process.^10^ Hence, while a broad set of instruments may boost communication, it does not necessarily help the process ‘move up the ladder’ for various reasons (cf. Fig. 1).

Instead, the case study comparison offers two other, less apparent elements for enabling a sophisticated exchange: efforts in trust-building and allowing a pluralist, inclusive panel of voices. Both are central for intercepting group dynamics and for enabling an unbiased exchange. Here, the event’s location also appears to be of particular importance: communication in the ambience of the stakeholders rather than in sterile conference rooms, which are more familiar to scientists, contributes to trust-building and eye-level communication. Besides the case studies from India and Colombia, the Isles of Scilly example shows outstanding efforts towards achieving these elements. Here, the researcher invested in public relations to introduce the local population to his project, and he interviewed members of the community individually while letting them decide on all ‘terms’ of the communication. A counterexample may be the Baltic Sea group discussions: some participants rebelled against the non-negotiated terms of the scientist-led evaluation approach; they thereby reclaimed some control over the process and managed to be heard. In the Bangladesh case study, efforts towards trust-building are less obvious, but the lengthy (and intimate) opening discussions conducted in local dialects may have acted as such.

Moreover, the comparison confirms that allocating necessary resources – time, in particular – is not only the most basic indicator but is also instrumental for achieving successful communication. The case studies that encountered the strongest drawbacks were those with the shortest time frame. In contrast, case studies that allocated more time typically received far better results.

The issue of planning is part of a bigger picture: as concluded in the Brazil case study, local stakeholders’ interests – mostly issues concerning their livelihoods – diverge from researchers’^11^ questions driven by the frontier of their fields. Hence, at best, projects should be co-designed with key stakeholders from the start, as the attempt to co-design research often challenges previously unreflected presumptions. While there is never enough time to seriously ‘co-design’ research with local and indigenous populations, even trying makes a substantial difference.

While most of our discussion focuses on how researchers can improve the process, it is crucial to remark that their hands are often tied by rigid, bureaucratic, and unappreciative funding policies. Especially in the Global North, grant allocation and budgeting practices by national research agencies often prove to be a roadblock by neglecting (or prohibiting) spending adequate resources on genuine stakeholder involvement [92]. Almost all case studies have expressed the concern that their funding (and the red tape behind it) actively prevented them from using sophisticated methods when communicating with local stakeholders or even investing in building relations with ‘research partners’. Currently, an increasing number of calls demand stakeholder interaction and interdisciplinarity on paper, but genuine efforts towards knowledge co-production and mechanism of co-design would be a political decision [43,93] that is neither met with interest nor the necessary resources.

## Stories of postcolonial moments

Postcolonial moments circumscribe a utopian communicative process, for which a lack of coherent (science) communication and the status-quo of knowledge generation need to be overcome. Therefore, the prospect of a method that structures these challenges along clearly defined indicators to generalise cross-culturally and create sameness [39] in understanding each other’s meanings opens a new and creative perspective.

Believing in the formative power of narratives, we selected three stories that broadly qualify for encouraging postcolonial moments. The following paragraphs provide additional background on communication experiences in Colombia, Bangladesh and the Isles of Scilly.

### In Bangladesh

*Even after all the preparatory work, we had difficulties making the local participants understand the concept of biodiversity, its value and its tangible impact on their livelihood. We, therefore, introduced the interactive half-hour session at the beginning of every discussion. The facilitators would start this session by building on familiar notions, using local dialects and referring to the participants’ own ecosystems. The participants were eventually able to catch up very quickly, as they found themselves in familiar territory. Thereupon, the group would become very interactive and ready to share central information with the facilitators. The interactive storytelling approach invigorated the participants and acted as an icebreaker. Still, facilitators worked continuously towards keeping the session as interactive as possible, using follow-up questions. As a result, the participants could grasp the concept of climate change and its impact on biodiversity; they completed their story, based on their own scenarios. The study bestowed a crucial lesson on the scientists: the gap between the scientific understanding of climate change/biodiversity loss and practical* ‘problems’ *of the marginalised community can only be bridged by understanding the community’s perspective and unearthing their knowledge-base, their way of problem identification, and their thinking on possible adaptive measures – using their very own language.*

### In Colombia

*The* ‘Future-proofing Conservation Project’ *in Colombia worked under the assumption that experiential learning is central to building capacity and understanding complex concepts. It involved creating spaces for stakeholders to develop and share ideas and discuss social values and the benefits from protected areas. Workshops with protected area staff and local stakeholders helped to explore key questions around ecological, social and economic values, and expectations for the future. This was the baseline to examine knowledge questions* (‘How will climate change affect these values?’) *and rules* (‘How can we prepare our institutions, and what have we learned from the past?’). *We adapted these workshops to local contexts and realities (i.e., times, needs and expectations). Crafting this common narrative helped to identify where and how to start while introducing climate change adaptation as a forward-looking policy, conducting planning and management, and determining practical tools to enable this. This facilitates identifying different or additional management to support the provision of benefits from protected areas. The narratives were broadly positive, centred on how people can explore their knowledge and values to improve protected area management in the face of unpredictable climate change*.

### On the Isles of Scilly (United Kingdom)

*There is not a single* ‘postcolonial moment’ *but a combination of various experiences during the fieldwork that had signs of mutual approximation and dialogue. The trustful relationship with research subjects allowed for an open and informal way of engagement with them that involved discussing and jointly reflecting on the research goals, questions and method. This engagement led to intense conversations on an equal footing. In some cases, they would concern the islands’ future and societal development in general. In other instances, they would lead to very critical and challenging discussions about the research’s key arguments, its approach and the role of human agency. Such discussions happened partly in rather intimate environments, such as at people’s homes, on a fishing boat or at their workplaces. Despite sometimes being highly challenging, they were always respectful and open. This exchange provided a crucial contribution to a* ‘postcolonial’ *perspective. It influenced the case study’s research approach, the interpretation of findings, and a more balanced representation of* ‘local voices’. *Moreover, it also affected the researcher’s way of looking at the world and his place as a researcher in a diverse community home to people ranging from residents with a long tradition of dealing with local challenges to newcomers with novel visions to external experts with specialist know-how.*

## Conclusions

More than 30 years have passed since climate change and the loss of biodiversity entered the global political agenda. Knowledge of these issues has grown considerably thereafter, but progress towards solving them has been meagre. Instead, ‘slow violence’ associated with the secondary effects of climate change and biodiversity loss, their mitigation and fast land-use change spread among local communities, especially in the Global South. These local communities are essential for data collection and policy implementation, but significant communicative gaps between researchers, practitioners and local communities often prevent success.

Therefore, this study has taken a closer look at the role of communication. At its core, it has focused on presenting, analysing and discussing eight case studies of communications between researchers and local communities, summarised in matrices (Tables 2, 3 and 4). Our study was eventually guided by the prospect of designing a method that structures the communicative challenge when addressing problems related to climate change and biodiversity loss along clearly defined indicators for good communication striving for postcolonial moments.

The rich panel of case studies, which crosses geographical and cultural boundaries and combines various instruments, approaches and degrees of communicative success, allowed us to make substantial learnings on how communication *is* and how it *should* be conducted. Case studies with an advanced approach towards communication (as measured by our indicators) had more communicative success and approached postcolonial moments, which allowed for disruptions of epistemic power relations towards the co-existence and discursive construction of alternative knowledge systems. In other cases, the communication processes yielded significant drawbacks, even leading to rebellious reactions among local stakeholders. Insufficient progress towards postcolonial moments became often visible in the form of a local disinterest in project results and a focus on quick solutions or monetary benefits. This would be the case, especially when the communication was not sufficiently de-hierarchised.

Furthermore, the case studies suggest a divergence between timing, complexity and (inner) effort towards (action for) making the communication more sophisticated. The indicators thus revealed an intrinsic logic and system of interdependency that does not correspond to the eventual timing within the project but follows patterns of rising complexity and inner efforts from the project team (planning, shaping the immediate communication, ex-ante scrutiny of the situation and sophisticating communication eventually). Therefore, although the case studies often presented a multitude of instruments towards shaping immediate communication, they rarely exerted more profound efforts towards scrutinising power relations or moving towards the equal co-production of knowledge.

This, however, contrasts with the necessities in the field. Whether the aim was to explore new domains, develop and implement solutions or disseminate and exchange existing knowledge, the case studies have shown that knowledge could only be co-produced by carefully creating de-hierarchised spaces for exchange. Although various (sophisticated) instruments in the practitioners’ toolboxes have proven to help deconstruct science, this analysis has shown that they are not always sufficient to remove barriers entirely. Instead, our results suggest that even simple instruments may suffice, while trust-building and allocating enough time for communication seem to be the more critical factors. Hence, instruments and communicating on equal footing hardly substitute for one another; instead, a combination of well-designed elements and an advanced awareness by scientists of their individual status and their postcolonial frame conditions – including approaches such as ‘critical whiteness’ [94] – are required.

We are aware of two limitations to our approach. First, and this applies to all case-study research, there is no way to ensure the generality of our results. However, we believe the substantial variation within our sample – covering different regions, approaches, teams, aims, instruments, resources and degrees of success – ensures a high validity. One active shortcoming is that our sample includes no development assistance project; however, we have no reason to believe that the results cannot be transferred to such communications. Second, our approach dichotomises the involved parties into an ‘external’ project team and local stakeholders. While this approach helps to focus on the communicative process, it underestimates the role of power relations within the project teams. These may, however, be able to provide explanations for some of the behaviour observed, such as the asymmetries found in the sophistication of projects. In fact, our observations suggest that diverging aims and power asymmetries within project teams may be as influential as the outsider–local gradient: who sets project parameters, who decides on budget allocation, who communicates and who is interested in what?

Proceeding to policy recommendations, we hope this article stimulates debate among financers about the importance of high communication standards in respective projects. Especially in the Global North, national research agencies’ adverse grant allocation and budgeting practices typically neglect (or even prohibit) financing anything but a narrow definition of cutting-edge research. Even research carried out by or with researchers from the Global South is often considered not ‘scientific’ enough by funding agencies and scientific publication outlets. Project activities that seemingly diverge from a colonialist (or even just conventional) approach, such as genuine stakeholder involvement, are often considered ineligible expenses. Yet, as this article and the vast body of literature we cited have shown, raising the bar of communication standards when interacting with local populations is not only a matter of development and ethics but also a prerequisite for excellent science. This structural deficit in research governance can also not be simply absorbed by the development sector, as their goals may not necessarily align with those of climate/conservation scientists. Currently, an increasing number of calls demand stakeholder interaction and interdisciplinarity on paper. Still, genuine efforts towards knowledge co-production and mechanism co-design are met with neither interest nor the necessary resources.

Therefore, and in line with Hulme’s ([80], p. 335) demand for a reorientation of research agendas towards a deeper understanding of the barriers towards sustainable lifestyles and their overcoming, our recommendation to policymakers is clear. We advise financing bodies to specifically require advanced communication styles in future research and policy implementation and alter grant and budget practices accordingly. A genuine cross-fertilisation between qualitative social sciences/humanities and quantitative approaches need to become the modus operandi in development-oriented research. Indicators such as ours or postcolonial moments themselves should become project deliveries to which adequate resources and time are allocated. We also encourage all researchers and development practitioners to insist on good communication practices – perhaps even consider our indicators when preparing and implementing fieldwork.

Combating climate change and biodiversity loss may first require changing how we, as scientists, development practitioners, and policymakers, think and talk about it.

## Figures and Tables

**Figure A1:**
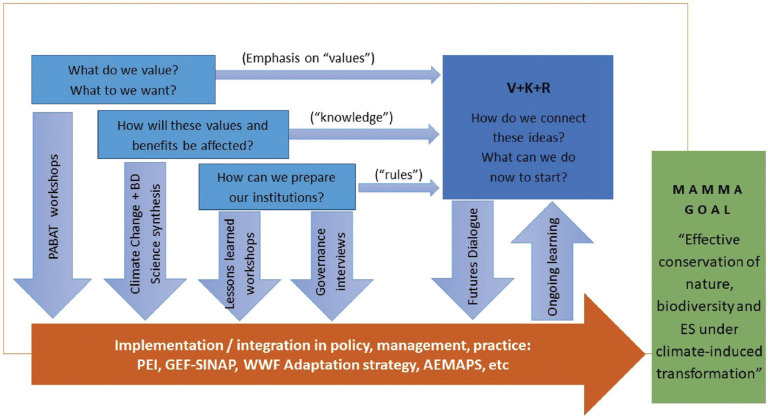
Conceptual framework used for the project in the Colombia case study [47].

**Figure A2:**
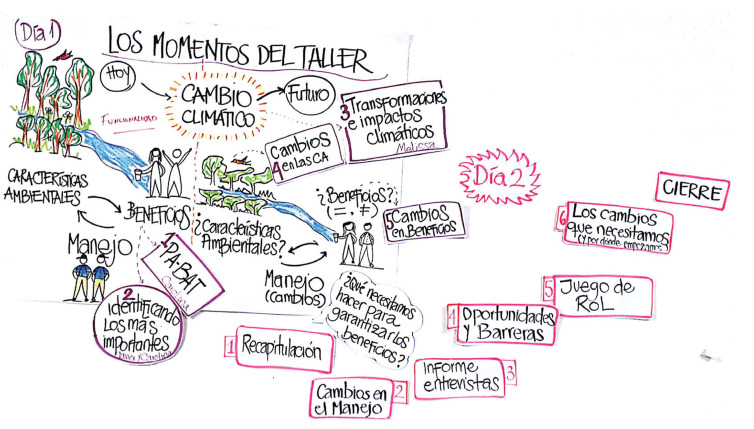
Cartoons designed by artists during the Colombia case study.

**Figure A3:**
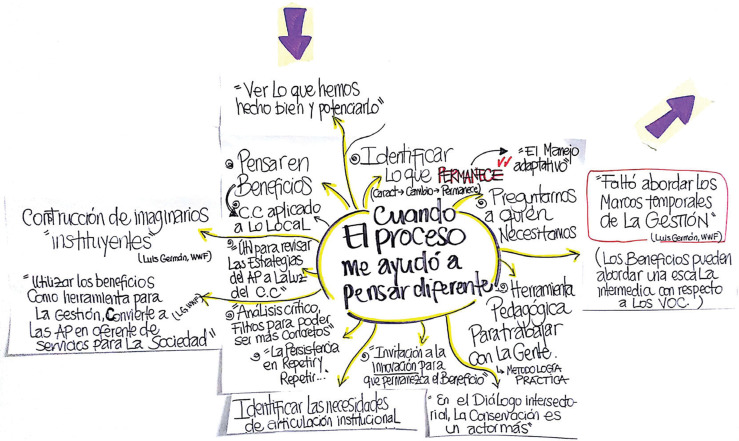
Illustration of the participants’ contributions and key findings in the Colombia case study.

**Figure A4:**
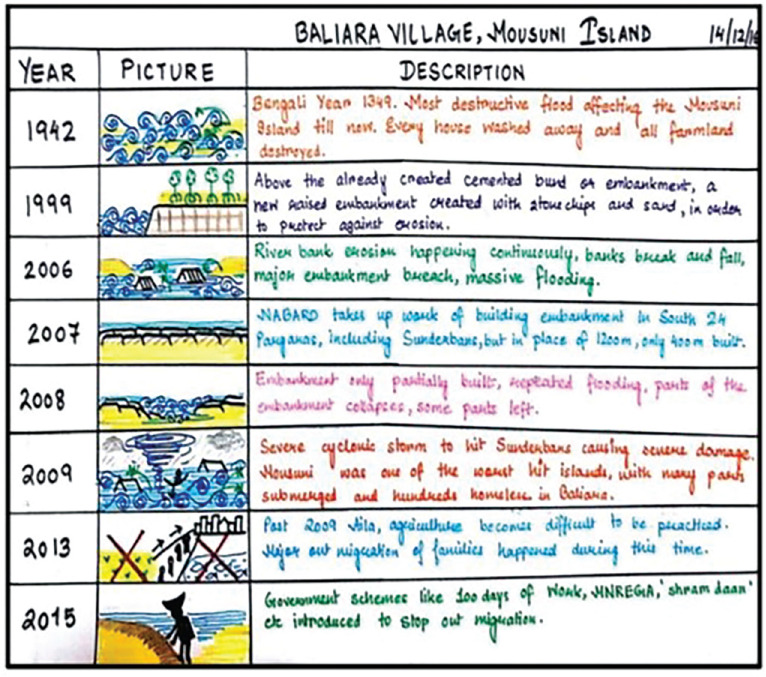
Timeline chart made by the villagers of Baliara in the Indian case study.

**Figure A5:**
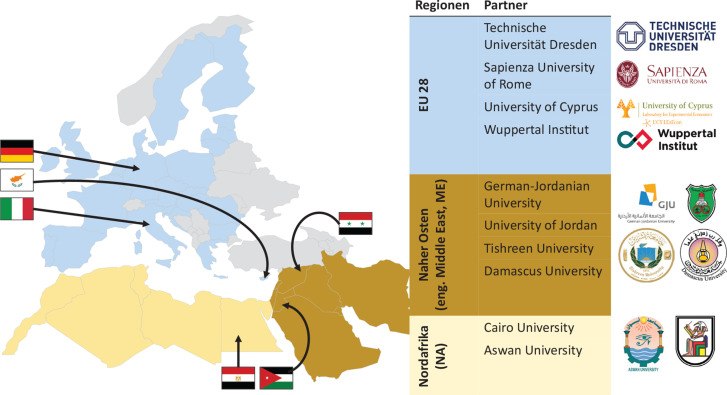
Overview of DESIRE project partners as part of the Egyptian case study.

**Figure A6:**
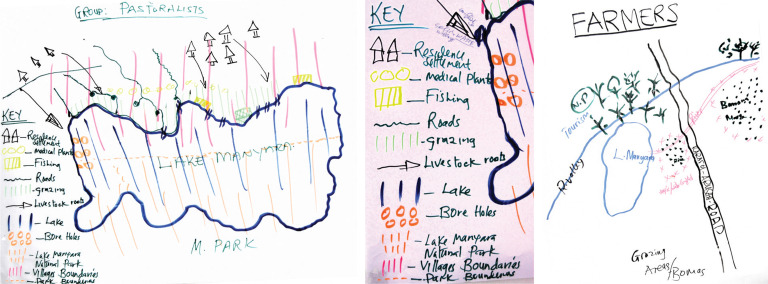
Examples of drawings from a community mapping exercise. Left: by representatives of the Maasai community (pastoralists). Middle: detail of the legend of the pastoralists’ map. Right: by representatives of smallholder farmers [54].

**Figure A7:**
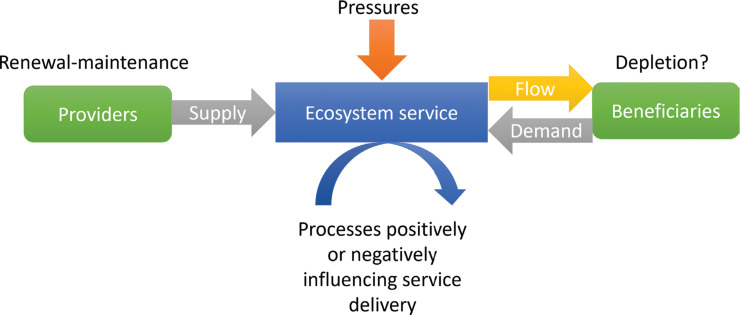
Scheme for focus groups to understand ES dynamics as part of the Tanzanian case study [54].

**Figure A8:**
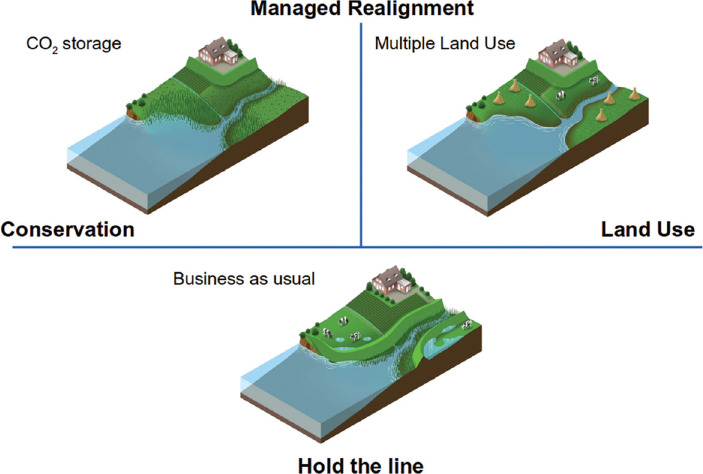
The three science-based COMTESS scenarios as part of the German case study.

**Figure A9:**
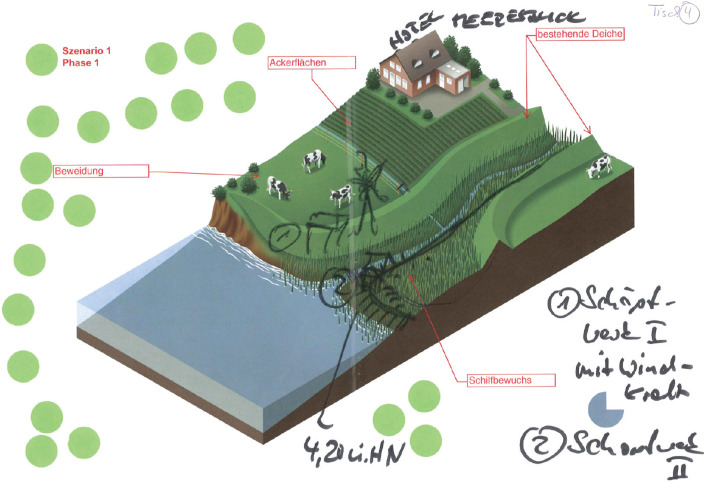
Dissident participants turn the given rules of evaluation around as part of the German case study.

## Notes

1 The spectrum can be seen in the participant structure of the Conventions of Biodiversity and Climate Conference of the People side events.

2 Although these local communication issues arise especially in the Global South, where most top-down measures for combating the consequences of climate change and biodiversity loss are implemented, we would like to emphasise that things are no better in the Global North. This is especially the case when it comes to science communication and the corresponding enforceability of science-based policies, as it can be seen, for example, European agricultural policy.

3 To this end, we limit the scope of analysis to the (expert) messenger and the message that is being sent, but we do not actively discuss the role of recipients (except for their feedback as to whether the communicative process was successful or not). Furthermore, we do not distinguish between different elements of climate change and biodiversity loss policies and projects but limit ourselves to investigating the communication process of a generic project.

4 Secondary effects are unintended and often neglected collateral damages that arise from policies or projects (such as, for instance, the disruption of fish reproductive cycles or the flooding of sacred sites caused by the building of a hydroelectric dam).

5 The postcolonial critique investigates the role of cultural forms and systems of knowledge in legitimising and sustaining asymmetrical power relations and the associated processes of exclusion and domination [83,84]. The foregoing reflections are thus aimed at problematising and calling into question the established concepts and interpretations of development, and critically reviewing our habits and ways of thinking and acting with regard to its discourses and practices, in light of the many forms of violence that development has generated in the lives of its putative targeted societies.

6 The case studies were selected from a pool of more than 20 case studies responding to a call by Ansari and Schönenberg.

7 The fact that Kayapó elders mostly focused on economic benefits could result from various factors. However, we hypothesise that insufficient inclusion in the research design was causative: As the researchers did not introduce local decision-makers to the concept of ‘deep carbon’ until shortly before presenting the results, the engagement was too simplistic and too late to generate interest beyond the monetary implications. Further research into why and when local decision-makers restrict their interest to economic benefits is necessary to gather evidence for this hypothesis.

8 In many cases, ‘local research partners’ would be a more adequate term, as the term ‘stakeholders’ remains vague and ambiguous. Nonetheless, we decided to use the latter, as almost all case studies used it.

9 The de-hierarchisation may, however, be a need less perceptible for the practitioners, as it transpires as soon as the fieldwork starts but actually implies a further sophistication of the process. Therefore, the indicator goes beyond the rather instinctive notion of the other indicators shaping the immediate communication.

10 Due to a lack of time, further methods of de-hierarchisation (e.g., group exercises for alternative eco-management options, a facilitation of local non-governmental organisation (NGO) support) could not be realised. This lack of a participative identification of local solutions is what may have cemented the impression that locals would have a solely passive role in the solution.

11 International development projects can have very similar issues. They are often derived based on the wider policy aims of the financing party, which do not need to be in line with the locals’ interests or troubles. Especially in the context of projects aimed at increasing awareness and action with respect to first-world environmental problems, the project goals can be entirely out of touch with the local world in least-developed countries.

## Open data and materials availability statement

Data sharing not applicable to this article as no datasets were generated or analysed during the current study.

## Appendix

### Appendix A (Amazon rainforest, Brazil) – communicating climate change: what’s the forest worth?

From 2011 to 2016, the German-Brazilian research consortium *Carbiocial* investigated the interdependencies of land use and climate change using the case of the 4476 km highway BR 163 crossing the Brazilian Cerrado and connecting this Brazilian hotspot of soybean and cattle production with the Amazon and its big river port Santarem. Universities from Germany, Austria and Brazil participated in this inter- and transdisciplinary endeavour. One example was the collaboration between the soil science project comparing soil carbon turnovers of the different land-use formats (mainly forest, fields and pasture) and the social scientists researching challenges and chances of social transformation for GHG-optimised land- and natural resource-management strategies. Jointly, the two sub-projects entered in collaboration with the local indigenous organisation at one of the research hotspots in the Northern part of the highway. The collaboration aimed at researching soil carbon stocks in the indigenous territories, the last pristine forest areas in a region with fast-changing land-use patterns and growing cattle and soybean cultivation [44]. The research team consisted of several soil science researchers (PhDs, master students and postdocs), two social scientists, a local well builder and several indigenous representatives. Jointly, they organised two expeditions to sample several smaller pits in the forest area in addition to a 10-m deep hole to analyse nutrients and carbon stocks. Part of the collaboration with the indigenous representatives was a presentation and handover of the research results afterwards. On the presentation day, we met with around 20 indigenous leaders from the diverse subgroups of the indigenous people and several members of the local representative institute the indigenous people had set up.

After some discussions among the scientific team, we decided to picture carbon as energy, starting the interaction by asking people where to find energy in their surroundings and taking the example of eating food for illustrating the transformation of carbon into energy. We then argued that energy would constantly change forms to refer to the carbon cycle and developed the narrative that balance was an ideal state for a cyclic system to be maintained. The effect of humankind disturbing the natural cycle and pushing things out of balance was a common narrative among indigenous representations of the present-day reality. This was confirmed when we asked if people perceived a disbalance in their environment, which was widely confirmed, and examples such as ‘drought’ and ‘fires’ were given by the indigenous leaders. This topic has acquired a sad continuity in the global news about Amazonia. To the natural scientists, it was imperative to stress that imbalance was the effect to be expected, which can mean heavy rains and droughts.

To contextualise the scientific findings, we decided to introduce the debate on carbon emissions and carbon emission trade, respectively its tool REDD+ to explain the utility of the collected data for the indigenous leaders. As had become clear from preparatory conversations with their local institute and conversations with the local NGO organisations, this had by far not been the first discussion on REDD+. Rumours were growing fast about money to be made, information and contacts to people dealing with these issues were considered necessary as the United Nations Conference on Sustainable Development (Rio+20) had taken place just in the previous year in Rio de Janeiro and brought global focus on the options for climate change mitigation in the Brazilian Amazon. Several Brazil-wide NGOs had previously given talks and workshops on REDD+ for the indigenous groups in and around the small Amazonian town. There was even an initiative to set up an indigenous programme called REDDindígena, a programme led by the Coordinator of Indigenous Organisations of the Amazon River Basin (COICA) to join payments for ecological services with participatory long-term land management plans set up by the indigenous communities. This is only one example of the attempts of global forest dweller representations to take the debate on the use and value of their territories back into their own hands.

One common critique of REDD+ mechanisms is the unfair negotiation resources between indigenous communities and international corporations acquiring certifications and the doorkeeper role that support organisations such as NGOs play in these negotiations. Therefore, a critical concern for us was to distance ourselves from the NGOs that were coming to the remote town over and over again, setting up projects involving participation and planning while providing few results. The related budgets and daily payments for participation in workshops were, of course, a coveted currency. In this heated field, we struggled to maintain a ‘neutral’, scientific identity by positioning ourselves as carriers of information and facts rather than opinions and plans.

We tried to explain the global REDD+ mechanism as a contract between who pays for the right to emit and somebody who concedes that right in exchange for monetary compensation. How much should be spent led to the question that seemed to be far more interesting than the theory: how much carbon was in the indigenous territory? We explained that the numbers presented were projections based on the samples, and also tried to explain how these results were reached (debating calculations via satellite images versus soil analysis), and even tried to make the argument that current calculations were considering way too short a layer of the soil – 50% of the soil carbon had been found in the layers below the 1 m layer that is taken as the basis for the common carbon stock calculation schemes [45].

From a scientific point of view, these debates were interesting. However, for our audience, questions of how the carbon price was determined, who decides it and whom to sell to naturally mattered much more. This hints at the often discussed problem that research interests do not always meet stakeholder needs – a challenging element of transdisciplinary projects [46]. In the end, a ceremonial handover of the results marked the end of the meeting. Later on, a YouTube video was produced to keep information about the collaboration alive, but it has still very few clicks as of August 2023. Enquiries during Q&A also showed the difficulties of linking the concrete with the abstract, for example, there were questions of whether emission trade had anything to do with selling dead leaves. What we suppose can be learned from this example about the global politics of climate change, is how difficult a debate ‘on equal terms’ about these questions actually is. On the one hand, there is an information overkill, including much fake news on the potential and reality of REDD+ mechanisms. On the other hand, information is always filtered as per the interest of the informer, which makes communities in remote areas with little access even less prone or empowered to participate in the global debates on climate change and possible mitigation.

### Appendix B (Colombia) – Co-producing and co-learning climate adaptation strategies in biodiversity conservation: lessons from Colombian protected areas

How do you conserve a glacial mountain when the glaciers are no longer there? How do you protect the habitat of an endangered species when the rainforest it depends upon transforms into a drier woodland? Conservation has traditionally been concerned with preserving, maintaining and restoring biodiversity, ES and special landscapes with scenic or cultural values for society. Climate change brings new and inevitable ecological transformations, where preserving, maintaining and restoring ecosystems may no longer be possible. In a rapidly changing world, where biodiversity in protected areas, and SES are under pressure, traditional approaches to conservation are fundamentally challenged. Managers not only need to learn new knowledge, but also new skills and ways of thinking as old certainties fall apart and new types of challenges emerge.

#### Context of communication

The Future-proofing Conservation project, based in Colombia, developed processes that enabled protected area managers to rethink the nature of conservation and management strategies in the context of climate change. The project successfully brought together different actors to ‘rethink’ protected areas management and governance and to move away from conserving particular ecological attributes (e.g., species) towards conserving values and benefits generated by SES managed through protected areas, while accommodating inevitable ecological changes.

This change sees climate adaptation focus more on how groups of actors – public, private, non-profit, community, business sectors – make decisions managing changes in the protected area and surrounding landscapes, rather than primarily on the biophysical aspects of climate change. This provides a better understanding of how decisions flow from on-site management actions (e.g., planning for declining water resources) to high-level objectives, such as maintaining a nationally representative system of protected areas.

This collaboration developed the ‘future-proofing process’ to help managers think differently about these complex challenges by considering future conservation goals and exploring ways to adapt protected area management.

#### Stakeholders involved in the communication process

Future-proofing conservation was a collaboration between academic partners (Australian National University, Commonwealth Scientific and Industrial Research Organisation), advocacy partners (World Wildlife Fund Colombia) and practitioner partners (Parques Nacionales Naturales Colombia), along with professional conservation advisers (Equilibrium Research) and a brokering organisation that sought to facilitate collaboration across sectors (The Luc Hoffmann Institute). The process was tested in two pilot sites, the Alto Fragua National Park in the Amazon Piedmont and the Otun Quimbaya Flora and Fauna Sanctuary, located in the country’s coffee growing regions.

#### Challenges in communicating the concepts

Adapting protected areas to climate change require changes in how we think about management. For the implementation team, the first challenge was translating academic language into something relatable to managers. This was increased by the need to translate between English and Spanish (Fig. A1). The collaborative process focused on overcoming some barriers that prevent action in the context of managing protected areas under climate change:

1. The science and narratives supporting conservation goals in protected areas, tend to focus on maintaining ecological attributes and prevent change. The language and concepts of accommodating ecological change are unfamiliar, and often not well received.
2. By definition, protected areas have a geographical restriction that limits discourse, governance and action to certain boundaries.
3. People think about climate change more as a technical problem, where scientific information is most relevant than a governance problem, where understanding how people make decisions that affect the future is critical.
4. Climate projections are often used as a primary input in conservation adaptation planning. Such scenarios can be disempowering for managers and limit their capacity to identify adaptation options.
5. This affects how people identify and use knowledge for making decisions and influence the rules for managing protected areas.

#### Overcoming the communication challenge

The process was a multi-step, interactive, dialogue-based series of activities that encouraged conservation practitioners to anticipate ecosystem transformation, anticipate potential impacts on benefits and values, and explore alternative management approaches.

By drawing together consideration of what people value about the protected areas, knowledge about possible ecological transformations based on climate projections, and institutional management options, participants identified what can be done now to prepare for uncertain futures.

In both case studies, the process included five stages, with varying levels of stakeholder involvement:

Stage 1: draw together local experience, knowledge about, and perception of climate change and adaptation through workshops with protected area managers and practitioners to learn together, and start building a shared narrative.
Stage 2: identify benefits from protected areas through a workshop with representatives from the local community, local stakeholders and managers from the protected area.
Stage 3: understand the decision making and governance context through in-depth interviews with managers and practitioners.
Stage 4: synthesise knowledge about potential ecological responses to climate change in the protected areas for academics, practitioners and managers.
Stage 5: a final stage called the ‘Futures Dialogue’, a workshop for exploration and reflection on ecological transformation, values, and management options, with managers, local communities’ representatives and practitioners.

The design and implementation of the workshops depended on the time, situation and context. Simple diagrams illustrating values with photos and words or phrases helped (see Fig. A3).

### Appendix C (Mousuni Island, India) – Communicating climate change in the Indian Sundarbans

The Sundarbans, one of the largest mangrove forests in the world, lies on the delta formed by a confluence of three rivers flowing into the Bay of Bengal, spanning the neighbouring countries of India and Bangladesh. It was declared as a World Heritage Site by UNESCO in 1985. The Indian Sundarbans comprises 106 islands, of which 52 are inhabited.

#### Context of communication

Climatic events such as cyclones and floods have adversely affected the Sundarbans, causing increased food insecurity and loss of livelihoods for islanders [48]. Consistent sea level rise and river-bank erosion result in loss of land, driving inhabitants to out-migrate. Saline water inundations following storm surges in a cyclone, such as the one caused by super-cyclone Amphan on 20 May 2020, have left thousands homeless, submerging villages for miles. While national and international stakeholders have been engaged in humanitarian relief work in post-disaster situations, communicating climate change processes and increasing awareness about the need to protect mangroves and biodiversity have been largely left to academics, NGOs and community-based organisations.

During 2016 and 2017, a primary survey for data collecteion was conducted on the Mousuni Island, one of the 52 inhabited islands in Indian Sundarbans. The sample included 120 respondents, selected through multiple stages of sampling, and data was collected in various ways, including focused group discussions (FGDs), key informant interviews and participatory rural appraisal (PRA) techniques. The primary objective of the research was to understand how water-related hazards such as riverbank erosion and rising sea levels increase the vulnerability of the island dwellers and result in decreased resilience to future climatic events. The survey aimed at understanding the role that the socio-economic characteristics of a community play in determining vulnerability to climate change and analyse the adequacy of different forms of institutional support available to the inhabitants.

#### Stakeholders involved in the communication process

The entire process of conducting interviews, collecting data and disseminating information happened in three stages with three stakeholder groups successively:

- Local NGOs: the NGOs, which have been working in Sundarbans for a long time and have built trust with the local communities, were approached and informed about the project, as having their support is crucial to reach the villagers.
- Local Government officials: the Indian democracy works with a three-tier system, in which local governments are the lowest tier of governance, and any climate policy intervention would need their sanction and support.
- Villagers: having the NGOs and local government members on board, the villagers were more open to conversing and attending seminars and workshops, in which information regarding climate change impacts and coping techniques were discussed.

The NGOs working in Mousuni are well informed about climate change and the resulting increase in sea levels, which cause a greater influx of saline water into the mangroves. This not only harms the mangroves but also decreases the productivity of the island soils. Regular sessions on salt-resistant farming practices and livelihood diversification are conducted by these NGOs, which have adequately informed the stakeholders about concepts on climate change and resilience, making it easier to de-construct and use climate-related terminologies during the research project.

#### Challenges in communicating about the concepts

The project was purely academic and aimed at collecting evidence to inform future policy decisions. Therefore, it was essential to emphasise to all stakeholders that there are no associated grants, benefits or allowances to be gained on taking part in the discussions. This is especially important for conducting ethical research in vulnerable contexts.

Years of living in abject poverty and the lack of institutional support in reaching sustainable solutions have made the islanders lose faith in the government. With limited agency and options available, some villagers have been felling trees to rebuild their own houses and embankments, causing a depletion of the mangrove cover. However, initiating discussions on these topics was challenging, as no one from the community wanted to take responsibility for such actions.

#### Challenge resolution techniques and rationale

Repeated discussions and assurance of no legal consequences helped identify the local lobbies involved in deforestation in the villages. Several awareness-raising sessions by using videos, documentaries and pamphlets in local languages helped create an atmosphere of understanding the need to protect the Sundarbans’ mangrove forests, one of the richest biodiversity hotspots in the world. A very effective technique to raise this community consciousness was conducting interactive sessions using PRA techniques, which involved two important exercises:

- Using the ground as a canvas, the villagers were asked to use locally available materials, such as sticks, stones and leaves, to denote different resources that the island is endowed with and then cross out the different resources now lost due to cyclones or floods.
- Similarly, the villagers were also asked to create a timeline of the different climatic events that have affected the island over the last 70 years. Mapping of different events and associated destruction helped in clearly visualising how depletion of the mangroves directly impacted the increased exposure of island inhabitants.

Results from both the exercises were then transferred on to paper and shown to everyone in the community to raise awareness about the importance of forests, the consequences of deforestation and its relation to increasing climate extremes, and possible solutions and options.

All the materials used for the exercises, including videos, questionnaires and resource materials, were selected to be location sensitive and relevant. While discussing concepts regarding the linkages between biodiversity loss (depletion of the mangrove cover) and increased intensity of climatic hazards, taking examples of individuals living within those communities and involving them in the exercises helped to establish trust, communicate openly and identify the actual challenges with which local policy implementation is faced.

The project recognised that local communities are aware and protective of their surroundings; however, locals may have to use natural resources to save themselves when it comes to survival. Thus, maladaptation practices often result from the absence of agency and options. A key takeaway from the project was the understanding that local community knowledge needs to be better documented and represented in broad climate change policy frameworks. This will enable better implementation of long-term resilience policies globally, with the local communities feeling more involved and accountable.

### Appendix D (Shyamnagar Upazila, Bangladesh) – Communicating grassroot stakeholders: climate change and biodiversity crisis in coastal Bangladesh

#### Context of communication

Bangladesh is a leading country with millions of people with vulnerable livelihoods dependent on aquatic systems that are impacted by climate and anthropogenic change and where fishing and aquaculture have evolved rapidly in the last decades with significant consequences for sustainability [49,50]. The country’s coast is vulnerable to a range of climate change impacts, from extreme events such as cyclones to slow-onset processes such as sea-level rise [51]. It has been hit by a number of high impact cyclones, causing extensive damage to life and property over the years. Events and processes such as cyclone, flooding, riverbank erosion, sea-level rise and salinity intrusion in the coast of the country have long been affecting the coastal margin by altering erosion rates, causing saline waters to intrude further inland, shrinking protective barriers and increasing flooding by cyclone and storm surges [52]. In addition, directly human-induced impacts from aquaculture, chemical pollution, overfishing and destructive fishing adversely impact fish biodiversity and catches, causing high fish seed mortality.

In this study, we explored the recent trends in aquatic ecosystems of the coast of Bangladesh by looking at its aquatic diversity, aquaculture practices and productivity, and a number of associated livelihood changes. We used FGDs and household surveys in the Shyamnagar Upazila (sub-district) in the Satkhira district, on the southwest coast of Bangladesh. The investigation covered the period of 2002–2012. It aimed at identifying the community perceptions on the changes in biodiversity in the aquatic production systems, their productivity and livelihood dependence, the main perceived impacts from climate and human activities, and the adaptation responses from the aquatic system livelihoods.

#### Stakeholders involved

The study included stakeholders emphasising and prioritising the interest of the community. It encompassed communications with *gher* (prawn/shrimp) farmers/labours, post-larvae (PL) collectors, crab fatteners and riverine fishers, fish traders, and earth (in *gher*) workers alongside people whose livelihoods do not directly depend on aquatic systems but on other pertinent sectors. The data and information were complemented with interviews with four key informants – a high school teacher, a female NGO worker, an Upazila fisheries officer and a Union Parishad (UP) member.

Through the authors’ experience and consultation with researchers, a reconnaissance survey was made to select the study area, study participants and the key informants, and build rapport with the study participants. The study area selected is a disaster-prone area. Some projects and programmes run by governmental organisations and NGOs have been ongoing mainly using a top-down approach and a few cases involving grass-root level stakeholders at different degrees. Nonetheless, the community was familiar with the terms ‘climate change’, ‘livelihood’ and ‘adaptability’, despite a somewhat fuzzy understanding. The terms ‘biodiversity’, ‘loss and value of biodiversity’ and ‘conservation’ were unfamiliar concepts for the stakeholders.

#### Challenges faced

Even after all the preparatory work, the facilitators had difficulties making the FGD participants understand the concepts of biodiversity, value of biodiversity, the loss of biodiversity, effects of loss of biodiversity on livelihood and the major causes of the loss of biodiversity. The participants also asked the data collectors/facilitators four major questions – (1) Why are you collecting these data/information?; (2) Are you planning a project/programme?; (3) Do you have any plan to slow down or stop the biodiversity loss in our area?; and (4) Do you have any plans to improve the socioeconomic status of the biodiversity dependent community?

#### Addressing the challenges

At the beginning of every FGD session, an interactive half-hour session was arranged to make the biodiversity concept familiar to the participant. The facilitators strategically started this session with a very familiar notion, using local dialect and on their very own ecosystems and flora and fauna of past and present. It did not take long for the participants to catch up, and, within 10 minutes, they were on familiar territory. Then, all participants became very interactive with much information on the biodiversity issues to share with the facilitators.

We used the simple way to deconstruct science: aquatic fauna. We asked the participants what they had in the past, what they have now, what they lost over the year, how this happened and what they considered to be the causes. In the beginning, we took an interactive storytelling approach using the local dialect and examples from the surrounding ecosystems. At every stage of that story, we asked the participants for their ideas and similar examples, before engaging them in a deeper dialogue. Eventually, the participants grasped the concepts of biodiversity and completed their story – based on their own scenarios.

During the FGDs, household surveys and meetings, the facilitators assured the participants that the study was not a development project but a research project of academic nature without monetary or material benefits for the participants involved.

#### Take-away-messages

There is a significant gap between the scientific understanding of climate change and practical issues for the disaster torn, poor and marginalised coastal community of Bangladesh. This gap can only be bridged through a thorough understanding of the community perspective and by unearthing their knowledge-base, that is, their way of problem identification and their thought process regarding the possible adaptive measures – using their language. This approach can be effectively and practically used in other similar locations where communities are affected by climate change issues and looking for a sustainable adaptive strategy.

### Appendix E (Aswan, Egypt) – The Aswan DESIRE Workshop on socio-economic impacts of RES in MENA countries

#### Background of case study

Countries of the Middle East and North Africa (MENA) are experiencing political, social and economic changes that are impacting the future design of domestic energy systems. With its sizable renewable resource potentials and the global trend toward decarbonisation, the region is gradually pivoting towards a post-fossil fuel economy. This transformation process is associated with various socio-economic opportunities and challenges. Against this background, the Erasmus Plus-funded project on the ‘**D**evelopment of higher **e**ducation teaching modules on the **s**ocio-economic **i**mpacts of the **r**enewable **e**nergy implementation’ (DESIRE) was launched in 2015 with ten different academic institutions from Europe and MENA countries (Fig. A5).

The project aims to create and implement teaching for MENA universities and support young professionals in evaluating the deployment of renewable energies (renewable energy sources [RES]) and energy efficiency measures in the context of socio-economic impacts. The project incorporated local stakeholder workshops with representatives from regional authorities, industries and academia to promote project outcomes and engage stakeholders in discourse about capacity deficits. The following summarises communication-related challenges that emerged during the stakeholder workshop organised in Aswan.

#### Stakeholders involved in the Aswan dissemination workshop

Public participation in sustainability research is important as a means of engaging stakeholders and facilitating solutions to societal challenges associated with accepting new modes of sustainable management. Dissemination workshops present a common form of such engagement. The Aswan dissemination workshop was held in February 2017 [53]. About 60 representatives from civil society organisations, local leaders, journalists and business owners participated in the workshop. The conference took place in the Helnan Hotel in Aswan. The agenda included talks on RES and their socio-economic impact, for example, health and job creation, in the morning session and a discussion on overcoming challenges associated with their introduction with all participants during the afternoon session. The workshop was well received at the local level, and the local news station reported on the workshop.

During the workshop, communication-related challenges of an organisational, cultural and conceptual nature were encountered.

#### Organisational challenges in planning the workshop

The scheduling of the dissemination workshop was decided upon by the DESIRE project team at their semi-annual meeting in the summer of 2016. The workshop was led by the Egyptian project partners. Due to the physical distance between partners, on-site support could not be provided in the preparatory stage. This hindered the European partners from assuming more active involvement in the event. For example, the workshop was held exclusively in Arabic, which posed a language barrier for the European partners. An active discussion among Arabic-speaking participants took place, which was well-received by those attending. A feedback survey indicated that more than 70% of attendees evaluated the workshop as ‘very good’ and ‘good’. However, more extensive coordination amongst the partners in the planning process would have been helpful in facilitating a more inclusive event that exploited the experience and expertise of all partners.

#### Cultural challenges in the execution of the workshop

The workshop served to make the public aware of the project objectives while also being used to facilitate input for the project, namely, the socio-economic benefits and associated challenges of distributed RES installations in local communities. Regarding the latter, the frontal nature of the workshop and lack of interaction, for example, discussions in small groups to pool a diverse set of experiences and input, limited the effectiveness of this portion of the event. While assumed to be very customary for the region, this more managerial format, in which most participants absorbed the information in a passive manner, hindered effective communication and exchange among the participants. This was partially caused by different perceptions regarding the workshop’s objective amongst the project partners. The local partners perceived the workshop as an opportunity to highlight the project outputs, while the European partners were more interested in utilising the workshop to advance project goals. The expectations of the workshop should have been addressed more explicitly beforehand.

#### Conceptual challenges in the exploitation of the workshop

As the socio-economic impacts of RES constituted the workshop’s main focus, a lack of a common conceptual understanding amongst participants limited its effective exploitation. Many participants from the MENA region had strong technical backgrounds. A narrow perspective limited to the technical deployment of RES that does not incorporate a shared interdisciplinary language hinders a contextualised discussion of socio-economic opportunities and challenges. This challenge is linked to the strong engineering focus in higher education systems in the field of RES in the MENA region. It would have been valuable to selectively invite a more diverse set of stakeholders to ensure a more comprehensive discussion of the project’s interdisciplinary character.

The set of communication challenges faced in the dissemination workshop in Aswan was diverse. The experience highlighted the need for awareness about these potential challenges, especially in an intercultural context, and making a concerted effort to address them explicitly at the outset of the project.

### Appendix F (Lake Manyara Basin, Tanzania) – ES as a rallying concept in multi-stakeholder workshops on biodiversity management and conservation around Lake Manyara, Tanzania

#### Where?

Lake Manyara is a saline lake in Northern Tanzania. It is the centrepiece of a national park teeming with iconic wildlife and a UNESCO Biosphere Reserve. The national park and surrounding areas provide much-needed revenue to the local economy through wildlife-viewing tourism, revenue-sharing schemes organised by the park authorities and co-management ranches. Despite these positive examples, the cattle of the Maasai pastoralists competes with wildlife and create overgrazing and erosion. Furthermore, irrigation agriculture depletes the scarce freshwater. Erosion, combined with heavy storm surges, provokes catastrophic mud- and rockslides, creating sedimentation of the lake, while prolonged periods of drought threaten the whole basin with water scarcity. This precarious situation is exacerbated by a fast increasing demography, climate change, the limited carrying capacity of this semi-arid region and complex governance. The multitude and diversity of stakeholders depending on ES provided by the biodiversity and aquatic systems of the Manyara Basin hold a variety of interests and opinions. Consensus on management decisions is uncertain.

#### Context of communication

We organised two participative workshops to achieve several outcomes: increased understanding of conservation policy and practice, capacity-building of stakeholders, co-creation of inputs for a decision support system and identification of research gaps.

#### Stakeholders in the communication process

A range of judgement elicitation methods were used in participatory workshops with representatives from authorities, NGOs, pastoralists, smallholder farmers and scientists [54]. This ‘evidence-informed’ approach inspired by Jahn et al. [13] is based on drawing information from the literature on the one hand, and by stakeholder knowledge about ES, on the other hand (focus groups and interviews). It aims at integrating the best available and socially robust evidence into decision-making. We used facilitated brainstorming within the focus groups by presenting our own work as an ice breaker and introduction, a collective stakeholder analysis (interest–influence matrix), problem/solution trees, drawings of community-specific maps of the region (Fig. A6), and prioritisation of ES. The flows of prioritised ES (Fig. A7) were then further documented, in different groups, according to their background. Climate change and erosion control were addressed by authorities and scientists, food from agriculture was documented by farmers, and water by pastoralists.

As an illustration, we present in Table A1 the main differences between maps drawn by the farmers and the pastoralists, respectively. This ‘community mapping’ is an excellent method to highlight different perceptions of the same landscape from different stakeholders in a participatory way. This can be important to understand the respective opinions and positions related to ES. Hence, formulating management recommendations for decision support, which are to a certain extent inclusive of the ‘world views’ of different land users, will contribute to reconcile or at least mitigate conflictual issues.

Moreover, collective field visits to projects facilitated by NGOs illustrated the challenges related to land use and natural resource requirements of farmers and pastoralists.

**Table A1.** Summary of main differences in the community maps drawn by farmers and pastoralists in the Manyara as part of the Tanzanian case study

Feature	Pastoralists	Farmers
Format	Landscape	Portrait
Point of reference	Eastern shore of Lake Manyara	Road (left and right of the road)
First five elements mentioned during oral presentation of the map	1. Eastern shore of Lake Manyara is drawn on top of the map (only map drawn in landscape format) 2. Lake Manyara 3. East of Lake Manyara: three coastal villages: Oldukai, Losilwa, Esilalei; borders between those villages are indicated; each village has its own access to LM 4. In between, also on eastern shore: natural vegetation from which people gather medicinal plants at a small scale 5. In between, also on eastern shore: fishing camps – use of dugout canoes	1. Main road Karatu–Arusha 2. Rift Valley 3. Lake Manyara 4. Small plots for rice, between Lake Manyara and the main road. In between: agriculture–water conflicts because the lake is depleting 5. South of Lake Manyara: grazing areas, bomas (a village called Esilalei) Crop fields are indicated larger than in reality. Big plantations are, however, not mentioned
Proportion of lake within map (estimation)	50%	8%
Elements only mentioned by one group	Medicinal plants, boreholes, village boundaries, better quality of grass at lakeside, livestock movements	Bananas, maize, rice
Conflict areas	Erosion at the sides of the lake, close to the shore of the lake, not at the grazing site. A dumping site next the lake (in the north)	Conflict between banana farms and rice farms Under Lake Manyara: grazing areas and bomas On the other side of the road: farming also (maize…) Water use conflicts
Missing elements	Agriculture	No legend for grazing area
LM sub-basin highlights	Highlighting the eastern grassland shores	Highlighting the road

#### Challenges in communicating

Biodiversity-related challenges are often presented as international and national targets and the indicators that go with them. This highly technical and standardised approach proposed by, for example, the Convention on Biological Diversity (CBD) or the Biodiversity Indicators Partnership [55] often does not match the limited awareness of these concepts among stakeholders involved in participatory workshops. Here we deconstructed biodiversity into ES as one main ‘rallying concept’: ES for a given protected area and its buffer zone, their perceived and observed trends, stakeholders and processes being affected by or affecting their delivery and value (Fig. A7). ES were approached through the benefits of understandable concepts such as land fertility, access to water or erosion control. Regulating ES such as water balance regulation, climate mitigation and air purification needed more explanation during the exercises. It is essential to find local examples that are close to the participants’ everyday lives. Besides the terminologies used, the communication process during the workshop may impact the comprehension of concepts and the willingness to participate. Small working groups communicating their results to the plenary were more efficient than direct communication in plenary where some people would not dare to talk or ask questions.

Moreover, it was important to have a simultaneous translation from English to Kiswahili as well as a local partner guiding the different group exercises, for example, explaining the different ecosystem services and making sure the group exercises were well-understood. Generally, our experience shows that mediation, moderation and facilitation of workshops in partner countries is often better understood by local communities when done by locally respected stakeholders, such as local civil society or a local expert, civil servant or academic. We identified hiccups with the communication towards local stakeholders during such workshops: expert jargon ill-adapted to the public, ill-defined target audiences that vary unpredictably between workshops, overly complex SES to tackle in a few workshops, or too high ambitions for ‘quick solutions’ and overly high expectations (‘we expect our livelihoods to be better after this workshop’, see video).

#### The next step: translating to decision support and policy

In the Manyara case, the focus on ES renders the language tangible and applicable for decision-making across governance levels, geographical and disciplinary boundaries. We usually co-produce policy briefs (PB) as the first step to policy outreach based on such participatory workshops (still to be done in Manyara). Cross-sectoral co-creation of knowledge, translated into a PB, incorporates local relevance and ownership to a higher decision level.

#### Take-aways

We plead for a ‘package’ of methods to customise communication amongst stakeholders of complex SES (e.g., stakeholder analysis, problem tree, community mapping, focus group, etc.). A rallying concept such as ES is key to mobilising stakeholders from various cultural and sectoral backgrounds in a constructive dialogue around biodiversity goals. More attention for biodiversity stakeholders will generate better-informed policies in which communities are visibly recognised and involved. Co-created PBs are, in that respect, promising tools. Multi-stakeholder participative workshops are also conducive for effectively translating the SDGs and (post-)Aichi CBD targets (Global Biodiversity Framework) to local communities and conservation stakeholders.

### Appendix G (Baltic Sea, Germany) – Dissidence and sabotage to redress scientific bias in communicating desirable coastal land management futures

The COMTESS project (sustainable COastal land Management: Trade-offs in Ecosystem Services - funded by the German Federal Ministry for Education and Research 2011–2015) modelled coastal land adaptation strategies on the German coast in the face of climate change. Its goal was to assess the potential of managed realignment (MR) to promote more resilient coasts compared to classical hard defence. MR restores natural coastal dynamics and buffers via the controlled removal of coastal dikes or their relocation inland.

The State of Mecklenburg–Western Pomerania is responsible for maintaining and upgrading first order dikes (that protect settlements). However, it is not legally obliged to maintain second-order dikes (that protect agricultural areas). MR on coastal agricultural land is per law possible but highly controversial at a societal level because it implies yielding land and control to the sea.

#### Stakeholders involved in the communication process

The modelling exercise included a participatory component in a four-steps approach:

1. Three science-based land-use scenarios were produced: that is, two based on MR: (a) In ‘CO_2_ Storage’, coastal land use is discontinued, and wetlands are restored, and (b) ‘Land Use Mosaic’ promotes ecological rich land uses that cope with temporary flooding, to be contrasted with (c) a control ‘Hold the Line’, business-as-usual scenario, where coastal dikes are upgraded and maintained.
2. The evaluation exercise was designed in a top-down manner. Selected experts were invited to discuss the congruence and plausibility of the three COMTESS scenarios and suggest alternatives for a fourth, ‘expert-based’ scenario. This round of consultation involved semi-structured interviews with experts from different perspectives on coastal defence, land planning policy, natural resource management, flood hazard rescue, conservation, agriculture and tourism.
3. The scientific team analysed the interviews searching for common trends that departed from the COMTESS scenarios to produce a fourth alternative ‘stakeholder-based’ scenario.
4. The four scenarios were evaluated by a bigger group of experts and members of the public during a World Café.

#### Challenges in negotiating how to communicate on/evaluate coastal futures

##### Climate change and its impacts were communicated as non-questionable

Effectively, no deconstruction of the concepts ‘climate change’ or ‘biodiversity’ was performed. The term ‘managed realignment’ was used carefully or avoided, as the notion of removing dikes generally raises negative associations. The term ‘land management’ was preferred to shift the focus away from coastal defence.

##### A participatory exercise fully framed and controlled by the scientific team

The project embedded little flexibility to incorporate stakeholders’ preferences:

- The scientists worked with narrow, pre-defined and non-negotiable assumptions related to coastal adaptation: that is, adaptation is needed and should involve MR. The ‘Hard Defence’ scenario was only envisaged as a control scenario to assess the gains and losses of the ‘real’ options based on MR.
- Experts could suggest alternatives but could not create their own scenario, as the scientific team had selected the most ‘promising’ expert contributions and collated them into a ‘stakeholder-based’ scenario.
- At the World Café, participants were to: (1) comment on visualisations of the four land-use scenarios and (2) mark the desirable outcomes (with green dots) or those to be avoided (with red dots). Participants were given an unequal number of green and red dots (3:1), the intent being to focus negative responses on one scenario (rather than all) to understand the stakeholders’ argumentation behind the rejection better.

#### Bottom-up response

Some World Café participants openly questioned the rationale of the project and evaluation exercise:

- The scenarios focused on specific aspects of a complex situation, which diminished their relevance.
- While a clear scientific prognosis for future impacts was requested, the future impacts visualised in the scenarios were questioned.
- Visualisations were abstract, difficult to understand intuitively, to differentiate or to relate to.
- The description of the scenarios steered participant’s opinion and, thus, evaluation.
- The evaluation method steered responses towards an apparent acceptance of MR.
- Group dynamics could influence the evaluation in either way.

Critical participants reclaimed some control over the process by:

- questioning the basic assumption on climate change impacts and the desirability of MR;
- producing their own future: for example, one where the highest standards in hard coastal defence would allow avoiding any climate change impacts completely;
- producing their own visualisation (e.g., by adding/removing elements);
- combining and distributing their red dots to dismiss all scenarios;
- not allocating any green dots on the proposed alternatives to visualise their refusal of these options.

By ‘sabotaging’ the prescriptive evaluation process, critical participants voiced their disagreement with the future options proposed by the project and made room for their own. While the project explicitly aimed at a participatory evaluation, it implicitly mainstreamed ‘managed realignment’. This inherent contradiction is common in top-down, nature-science dominated modelling projects, where scientists feel ‘forced’ into a co-design approach (e.g., by funding requirements). Collaborating social scientists often find themselves in an impossible conundrum: they must deliver a participation process when effectively they are expected to manufacture ‘societal’ legitimation. Fortunately, in our case, ‘dissident’ participants refused to endorse the project’s implicit strategy.

This case study provides valuable lessons towards true transdisciplinary modelling. Projects should:

- recognise and acknowledge perception, preference and rationalisation gaps between science, policy and society;
- accept the value of and accommodate departures from science-based assumptions;
- yield control in the research process to enable true exchange and co-learning.

### Appendix H (Isles of Scilly, United Kingdom) – Communicating climate change: fieldwork experiences from the Isles of Scilly

#### Context of communication

The project on social capital, resilience and adaptation to climate change on the Isles of Scilly took place between 2013 and 2016 [56,57]. The project’s objective was to analyse the role of social capital and community resilience in the context of climate change and was carried out by one PhD researcher from the University of Hamburg. On the Isles of Scilly, climate change manifests itself mainly through storm surges and sea-level rise, associated with coastal erosion, flooding, damage of coastal infrastructure and disruption of transport. Due to the relative isolation and peripheral setting of the five islands that constitute the Isles of Scilly at 45 km off the southwest coast of the United Kingdom, I (J.P.) was interested primarily in how self-organised community action can help to deal with the challenges posed by climate change. After a first preparatory field visit including a couple of scoping interviews and field observations in December 2013, the first full fieldwork phase of my project (February/March 2014) took place when the strongest storms in recent decades hit the islands, followed by a second fieldwork phase in July 2014 and wrap-up/discussion phase in September 2014.

My research consisted of a mixed-methods approach. The quantitative part included a survey entitled ‘Communities and the sea’. Besides a section on indicators of social capital, the survey included a section with 11 questions about people’s general perception of climatic change, as well as specific experiences of coastal risks, coastal management and participation in coastal protection. The qualitative part of the research involved participant observation, expert and stakeholder interviews. In addition, media analysis was undertaken by systematically searching local magazines and websites.

#### Stakeholders involved in the communication process

The different stakeholders’ understandings and perceptions of climate change, its impacts on the community, and potential adaptation measures were central concerns of the project. Stakeholders in the study included, amongst others, the local population, media, local council, non-governmental organisations and the landholder.

Climate change is an issue of which the local stakeholders were very aware. Due to their low-lying topography, the media has been dubbing the islands the ‘Maldives of the Atlantic’, and the local council employed a ‘climate change officer’ at the time of research. A local NGO was raising awareness about climate change concerns within the population and the local council. The local administration considered plans to develop wave energy instead of relying on a coal-fired power plant. Many islanders were aware that the archipelago was one island once and that incremental sea-level changes over the past millennia created the archipelago’s current shape. Notably, the publication ‘Exploration of a drowned landscape’ [58] made this fact commonly known. Also, indicators of submergence, such as coastal archaeological findings, submerged fields and artefacts, reveal the long history of sea-level rise. Therefore, exposure to climate-related hazards, such as storm surges and sea-level rise, make up part of the islands’ identity.

#### Challenges in communicating about the concepts

The coincidental timing of my research during the severe storm events helped facilitate dialogue about climate change with locals. Building on these hazards as a proxy for climate change was both reasonable and unavoidable. However, the general risk perception might have been overly shaped and potentially exaggerated due to those recent experiences.

A certain scepticism towards external ‘experts’ (e.g., from the UK Environment Agency) was often raised during interviews. While I did not perceive that people were sceptical towards my research, this attitude may have influenced how people talked to me and what they revealed to me about their risk perception and community life.

The diverse group of stakeholders involved different experiences and conflicting interests concerning the community and the environment and its stewardship. For example, the conservation and heritage aspect, involving minimal interference in the landscape, was pushed forward, especially by landholders and NGOs. On the contrary, residents and businesses were concerned with employment, tourism, habitability and coastal protection on the islands.

#### Challenge resolution techniques and rationale

An essential element to overcome a biased perspective due to seasonal weather conditions was to spread the fieldwork over various seasons (i.e., autumn, winter, summer) when different people live on the islands and weather conditions are different.

I approached the local people through their main local communication channels early in the research design. To overcome the scepticism towards me as an external researcher, I announced my research project via the local Radio Scilly, the main news website ScillyToday, and talked about my activities in a follow-up interview with the radio station. Therefore, when I started my research activities, many islanders were already aware of my intentions and very open to contribute their perspectives.

In order to gain local people’s trust and acknowledge their perception of climate concerns as a ‘counter-narrative’ to the influential voices by council, landholders and NGOs, participant observation, personal distribution of the questionnaires, and ‘walking interviews’ were essential – that is, letting people show me their sites of interest concerning potential climate change hazards, on their own terms.

Generally, explicit reference to climate change was only mentioned in expert interviews and interviews with stakeholders directly involved in adaptation planning. With other stakeholders and laypeople, climate change was deconstructed in terms of climate-related hazards and impacts, such as storm surges, shoreline change, erosion and flooding.

Finally, a report back of the research results and my interpretation of them to the community served as a tool to critically reflect on my findings and involve different stakeholders again.

#### Conclusions

In conclusion, three elements were crucial for communication during the research project:

1. A critical understanding of local awareness of and experiences with climate concerns and narratives about the local environment (e.g., sea-level rise) was key for deconstructing climate change and developing my research tools.
2. The combination of including different voices and over different seasons was a critical element to reduce a biased representation of climate risk – both concerning the influence of dominant stakeholders and seasonal variability.
3. Being transparent about the research approach, its aims and results by using the most common local communication channels greatly increased local trust and participation.
4. Participant observation and open-minded informal exchange with local community members allowed for building trust, an in-depth understanding of the diversity of local concerns (also apart from climate change) and the representation of otherwise marginalised voices.
